# A scoping review of importation and predictive models related to vector-borne diseases, pathogens, reservoirs, or vectors (1999–2016)

**DOI:** 10.1371/journal.pone.0227678

**Published:** 2020-01-15

**Authors:** Tara Sadeghieh, Lisa A. Waddell, Victoria Ng, Alexandra Hall, Jan Sargeant

**Affiliations:** 1 Public Health Risk Sciences Division, National Microbiology Laboratory, Public Health Agency of Canada, Guelph, Ontario, Canada; 2 Department of Population Medicine, Ontario Veterinary College, University of Guelph, Guelph, Ontario, Canada; 3 Centre for Public Health and Zoonoses, Ontario Veterinary College, University of Guelph, Guelph, Ontario, Canada; Faculty of Science, Ain Shams University (ASU), EGYPT

## Abstract

**Background:**

As globalization and climate change progress, the expansion and introduction of vector-borne diseases (VBD) from endemic regions to non-endemic regions is expected to occur. Mathematical and statistical models can be useful in predicting when and where these changes in distribution may happen. Our objective was to conduct a scoping review to identify and characterize predictive and importation models related to vector-borne diseases that exist in the global literature.

**Methods:**

A literature search was conducted to identify publications published between 1999 and 2016 from five scientific databases using relevant keywords. All publications had to be in English or French, and include a predictive or importation model on VBDs, pathogens, reservoirs and/or vectors. Relevance screening and data characterization were performed by two reviewers using pretested forms. The data were analyzed using descriptive statistics.

**Results:**

The search initially identified 19 710 unique articles, reports, and conference abstracts. This was reduced to 428 relevant documents after relevance screening and data charting. About half of the models used mathematical techniques, and the remainder were statistical. Most of the models were predictive (87%), rather than importation (5%). The most commonly investigated diseases were malaria and dengue fever. Around 12% of the publications did not report all the parameters used in their model. Only 29% of the models incorporated the impacts of climate change.

**Conclusions:**

A wide variety of mathematical and statistical models on vector-borne diseases exist. Researchers creating their own mathematical and/or statistical models may be able to use this scoping review to be informed about the diseases and/or regions, parameters, model types, and methodologies used in published models.

## Introduction

Vector-borne diseases (VBDs) are diseases spread by arthropods, which transmit pathogens between humans, reservoirs, and fomites, such as dengue (spread by mosquitoes) [1], Lyme disease (spread by ticks) [2], and leishmaniosis (spread by sand flies) [3]. As a result of globalization and climate change, certain VBDs will likely be identified in regions where the disease is not endemic (a disease that is not in constant presence within a geographic area or population group) [1, 2, 4], due to modes of disease importation such as human travel and transport, animal migration, environmental changes to habitats, and vector range expansion [4–6]. While vector-borne pathogens are occasionally introduced into non-endemic regions, other factors are required for a pathogen to become established, such as the existence of local animal reservoirs, susceptible human population, and competent vectors which can sustain the transmission of the pathogen where introduction occurred [4]. As average temperatures become warmer and precipitation changes as a result of climate change, vectors and reservoirs are expected to move into, and become established in areas that were previously unsuitable [1, 7]. Range expansion of vectors has already been observed, as in the case of the tick that transmits Lyme disease, *Ixodes scapularis*, which has moved northward through the United States (US) and into Canada [8].

Mathematical and statistical models can be used to predict the spread of VBDs, pathogens, reservoirs, and vectors. Mathematical models include a single equation or set of equations, which simulate or explain a system, and/or forecast future behaviour of that system; whereas, statistical models use methods of modeling which involve the compilation, analysis and/or interpretation of datasets (e.g. regressions). Both types models can provide information for public health and medical decision-making, for advocacy and program development targeting these diseases, and for prevention and control activities [9]. Two specific types of models are predictive models and importation models. Predictive models forecast the distribution or spread of a disease, pathogen, reservoir and/or vector over time, and have been used to model the effects of climate change on chikungunya transmission in Europe [10], as well as on the increasing range of vectors, such as *Aedes albopictus* on many continents globally [11, 12]. Importation models investigate the introduction and/or movement of a disease/pathogen via a reservoir and/or vector from an endemic region to a non-endemic region, and have been used to investigate Zika virus [13], and dengue [14] introduction and transmission in different regions via global air travel.

In the rapidly evolving field of mathematical and statistical models, there is a lot of variability in the methodology and parameters used, thus a scoping review was used to compile and characterize the relevant modelling literature in order to understand the research conducted in the area. Scoping reviews are a method of knowledge synthesis that maps the existing literature on a broad topic, using a methodological framework to make it reproducible and updateable [15].

Our objective was to conduct a scoping review to identify and characterize predictive and importation models related to VBDs that exist in the global literature. Models that specifically investigated the impact of climate change on VBDs, pathogens, reservoirs, and vectors were highlighted, as climate change is one of the many factors influencing VBD importation, transmission, and spread [1, 7].

## Materials and methods

This scoping review followed the methodological framework for scoping reviews [15]. The protocol (S1) for this scoping review was developed by the research team, and contains definitions, search parameters, and tools used to conduct the scoping review. Most sections of the protocol were completed *a priori*, except for the data charting form, which underwent additional modifications after relevance screening was initiated. The protocol was not time-stamped or registered. The reporting of this review followed the Preferred Reporting Items for Systematic Reviews and Meta-Analyses Extension for Scoping Reviews (PRISMA-ScR) (S2).

### Eligibility criteria

English and French documents published from January 1, 1999 to December 20, 2016 were eligible for inclusion. West Nile virus was first introduced to the US in 1999, and after this time predictive disease modelling was increasingly conducted due to a combination of improved computing power and available expertise [16]. Models created and published prior to 1999 have been excluded from this scoping review as it was decided there would be little to learn from this literature that is not still reflected in more current models. The document types eligible for inclusion were journal articles, grey literature describing primary research (original models), and theses/dissertations. Reviews, editorials, and commentaries were not eligible for inclusion because these document types do not typically include primary research/original models.

### Information sources

Databases with particular relevance to public health and environmental studies were selected. These databases included 1) MEDLINE (via PubMed); 2) Scopus; 3) Web of Science Core Collection; 4) Global Health (via Ovid); and 5) GreenFILE (via EBSCOhost). Searches for grey literature were conducted by searching via Google until there were five pages of results in succession without a relevant citation identified. Searches were conducted on December 20, 2016.

### Search

Searches were conducted using the following algorithm: ((import OR imported OR importation OR importations OR introduction OR transmission OR spatial OR spread OR expansion OR expand OR spatiotemporal) AND (model or modeling or modelling OR forecast OR predict OR prediction OR projection OR "risk analysis" OR "risk assessment" OR "pathway analysis" OR traveller OR travellers OR traveler OR travelers) AND ("vector borne" OR vectorborne OR vector OR vectors OR reservoir OR mosquito OR flea OR fly OR tick OR zoonotic OR zoonoses OR zoonosis OR viremic OR viraemic OR viremia OR viraemia OR bacteremic OR bacteraemic OR bacteremia OR bacteraemia)) NOT (protein OR transfusion).

Identical terms were used for each database; however, formatting was modified to follow the requirements of the specific database. Other than the date of publication (January 1, 1999 –current), there were no other restrictions on the search.

Search verification was conducted by manually searching the reference lists of ten randomly selected relevant papers for titles, which may be relevant to this scoping study [10, 14, 17–24]. All papers identified by the verification went through the same process as the papers initially found. Search verification was considered completed after the tenth paper, as new potentially relevant publications were no longer being identified in the final three reference lists searched.

The search results were compiled and duplicates were removed in EndNote (Clarivate Analytics, Philadelphia, United States). The results were then exported to DistillerSR (Evidence Partner, Ottawa, Canada), a web-based systematic review management program, for further de-duplication and management of the scoping review process.

### Selection of sources of evidence

Relevance screening included the evaluation of titles and abstracts to identify citations that may be relevant to the scoping study. Relevant publications included those that were written in English or French; that focused on a VBD, reservoir, vector, or pathogen; that included a predictive or importation model in their methods; and that originated from academic journals, theses, and grey literature sources if their methods included a novel and original model. Two reviewers independently evaluated each citation using a relevance screening form created for this review. Conflicts were resolved by consensus between the reviewing pair or, if necessary, by consulting with a third reviewer. Two pre-tests were conducted with five individuals using 50 of the identified citations in order to evaluate the relevance screening questionnaire for clarity, and assess consistency between reviewers.

### Data charting process

The goal of the data charting process was first to confirm that each full text paper was relevant to the study question using identical questions from the prior relevance screening stage, and then to characterize pertinent information required to appropriately describe each predictive or importation model. A data charting form (in protocol, S1) was created for this phase and uniformly implemented on each relevant publication by two reviewers working independently. Conflicts were resolved by consensus between the reviewing pair, or, if necessary, by consulting with a third reviewer. A pre-test was conducted with three individuals using ten citations in order to evaluate the data charting form for clarity, and assess consistency between reviewers.

### Data items

Topic areas of interest to this review included article characteristics, such as language and year of publication; model methods, such as model type (predictive model, importation model, or both), model class (mathematical model, statistical model, or both), subject of the model outcome (e.g. vector, human, or reservoir), outcome measured (e.g. probability of presence of a vector, number of infectious humans, reproductive number), region modelled, the importation pathway if importation was modelled (e.g. via vectors, reservoirs), parameters used in the model, whether or not a projected climate model was used, and if so, which climate scenario was used, as well as diagnostic methods used for the model (e.g. verification, validation, sensitivity). A select number of the important terms and definitions are listed in Table 1. The data charting form includes all associated definitions (S1).

**Table 1:** Key terms and definitions used throughout the scoping review.

Term	Definition
Importation model	Mathematical and/or statistical models used to predict the introduction and/or establishment and/or movement of a disease, pathogen, vector and/or reservoir via a reservoir, vector, human, fomite and/or non-reservoir animal from an endemic region into a non-endemic region An importation model can also be a predictive model if the movement of the pathogen is shifting from an endemic to a neighbouring non-endemic region; however, if the geographic spread occurred over two non-neighbouring regions, the model would be considered solely an importation model.
Predictive model	Mathematical and/or statistical models used to forecast the temporal and/or geographic spread, and distribution of a disease, pathogen, reservoir or vector.
Mathematical model	A single or set of equations which simulate or explain a system, and/or forecast future behaviour of that system [25]
Statistical model	Methods of modeling which involve the use of equations to compile, analyze and/or interpret existing datasets (e.g. regressions) [26]
Verification	Determining the degree to which the model output accurately represents the logical framework conceived by the modeller [27]
Validation	Determining the degree to which a model is an accurate representation of the real-world system the model is simulating [27]
Sensitivity	Determining the degree to which the model output changes when changing the input parameters (within values dictated by literature and common sense) [25, 27]
Climate model	A set of mathematical equations which simulate a climate system [28]
Representative concentration pathways (RCP)	Possible climate futures described via greenhouse gas concentration trajectories. Currently four are used, with RCP2.6 being the least projected rise in greenhouse gas concentrations, and RCP8.5 being the most (RCP2.6, RCP4.5, RCP6, RCP8.5) [28]
Special Report on Emission Scenarios (SRES)	Previously used future climate scenarios based on global, regional, economic, and environmental factors. It includes the following scenarios: A1 (A1FI, A1B, A1T), A2, B1, B2. RCPs replaced SRES in 2014 in the 5^th^ Intergovernmental Panel on Climate Change (IPCC) assessment [28]

### Synthesis of results

Following the charting of all eligible full-text articles, the extracted data were exported into a Microsoft Excel (Microsoft Corporation, Washington, United States) spreadsheet. Data cleaning, descriptive statistics, and summarization were conducted using Microsoft Excel (Microsoft Corporation, Washington, United States) and StataIC 14 statistical software (StataCorp, Texas, United States).

## Results

**Fig 1:**
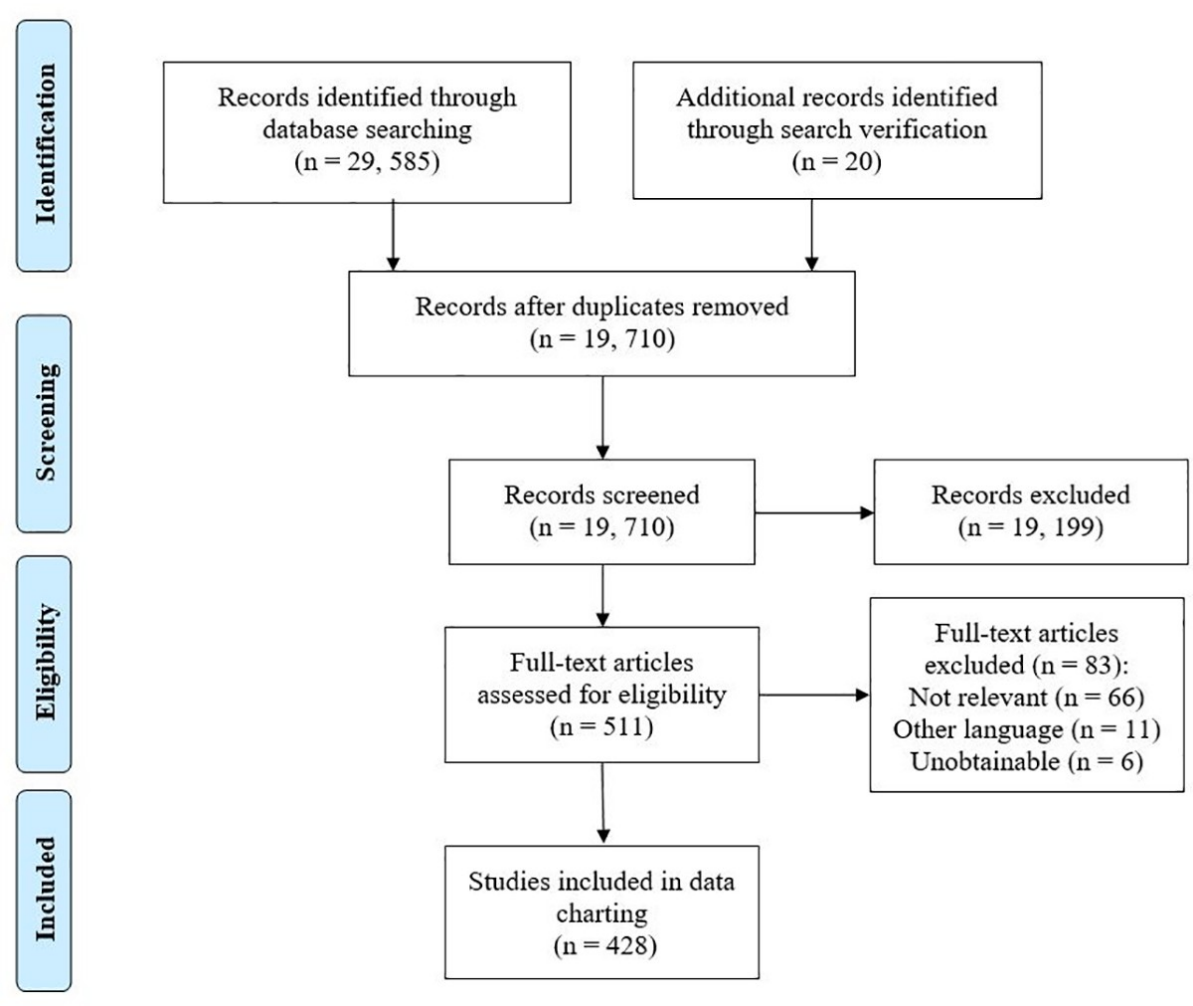
PRISMA diagram. PRISMA diagram depicting the flow of captured publications through the eligibility and inclusion process.

The initial search was conducted on December 20^th^, 2016 and yielded 29, 605 citations (Fig 1). Following relevance screening, the full texts of 511 publications were assessed for eligibility, of which 83 were excluded; 428 publications remained in the review for data charting and analysis. All included publications were in English, except one which was in French; however, 11 of the 428 potentially relevant publications were excluded due to language. A full list of the 428 included publications can be found in S3.

**Fig 2:**
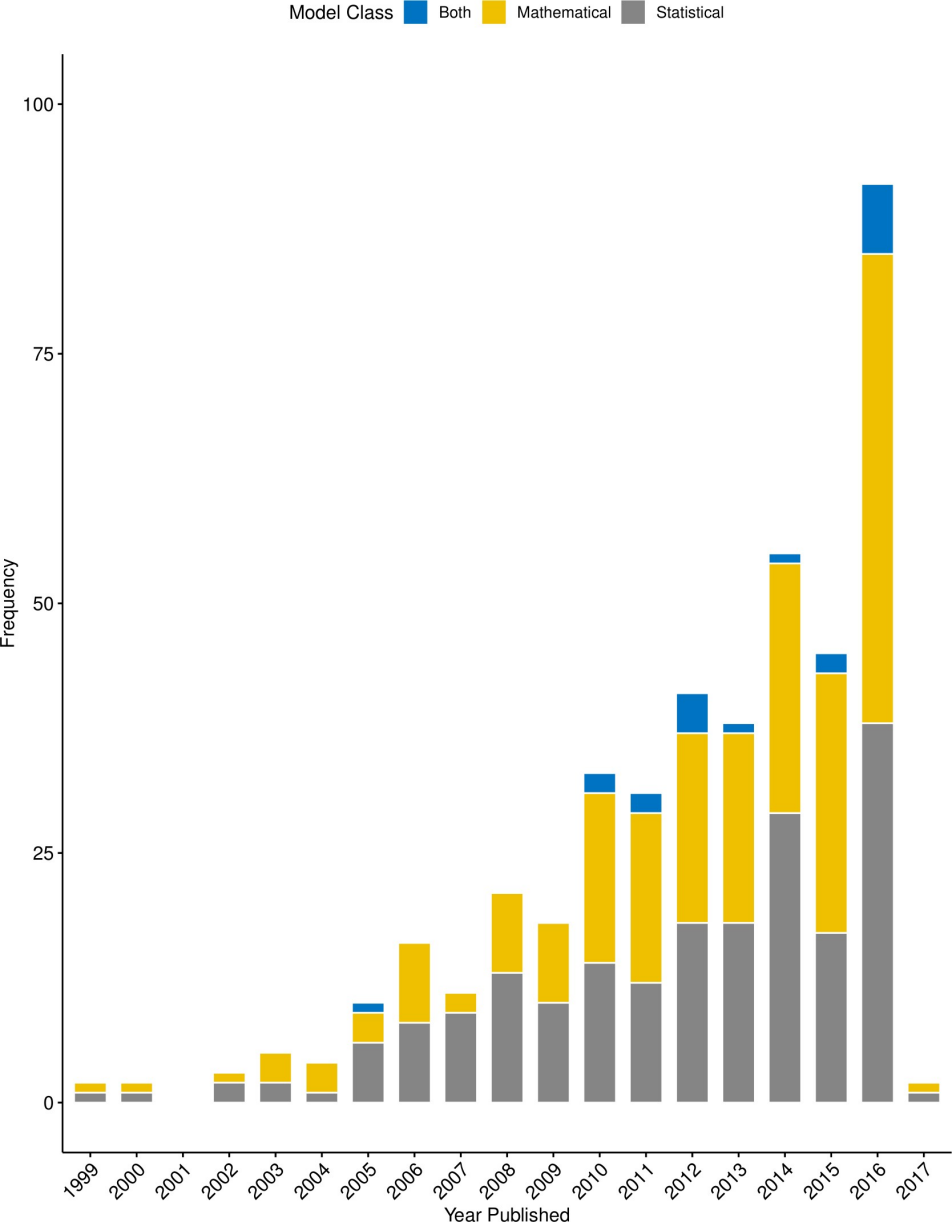
Frequency of the 428 relevant publications by year. Frequencies are separated by model class: mathematical and statistical (captured two pre-published journal articles for 2017).

Most of the 428 relevant publications were journal articles (95.8%, n = 410), while 2.6% (n = 11) were conference papers and 1.6% (n = 7) included other grey literature, such as government reports. The “other” category for grey literature included reports and chapters in published books. About half of the publications were published after 2013 (Fig 2).

The relevant models included in this scoping review were evenly split between mathematical models and statistical models (Table 2). Most of the models were predictive (87%, n = 374), and about half of the predictive models used mathematical methods (45%), while the other half used statistical methods (50%). The remaining 5% used both methods. Five models (1%) only focused on importation and used solely mathematical methods, and 49 (11%) used both predictive and importation model methods (Table 2).

**Table 2:** Frequency of the characteristics of the models including in the scoping review from 428 relevant publications.

Characteristic	Number	Percentage (%)
Model type		
Predictive	374	87.38
Importation	5	1.17
Both	49	11.45
Model class		
Mathematical	208	48.60
Statistical	200	46.73
Both	20	4.67
Disease or pathogen investigated*		
Malaria	70	15.98
Dengue fever	60	13.70
West Nile fever	31	7.08
Rift Valley fever	18	4.11
Schistosomiasis	16	3.65
Lyme disease	13	2.97
Chikungunya	12	2.74
Plague	11	2.51
Zika	10	2.28
Leishmaniosis	9	2.05
Chagas disease/American trypanosomiasis	6	1.37
Crimean-Congo haemorrhagic fever	2	0.46
Japanese encephalitis	2	0.46
Sleeping sickness/African trypanosomiasis	2	0.46
Yellow fever	1	0.23
Not applicable (investigated a vector or reservoir)	146	33.33
Other	27	6.16
Not specified	2	0.46
Region modelled*		
North America	95	18.48
Africa	94	18.29
Asia	84	16.34
Europe	72	14.01
Central America/South America/Caribbean	65	12.65
Australasia and New Zealand	24	4.67
Russia	11	2.14
Oceania	5	0.97
Global	25	4.86
Not reported/specified	39	7.59
Model scale		
Local	70	16.36
Regional	140	32.71
Country	76	17.76
Multi-country	86	20.09
Global	25	5.84
Unspecified	31	7.24
Subject of model outcome*		
Vector	278	50.27
Human	206	37.25
Reservoir	65	11.75
Other	4	0.72
Importation Pathway used, if relevant*		
Human	31	49.21
Vector	16	25.40
Reservoir	14	22.22
Fomite	2	3.17
All parameters reported		
Yes	348	81.31
No	51	11.92
In Supplement	29	6.78
Time/space in model (n = 428)		
Temporal only	115	26.87
Spatial only	108	25.23
Temporally-spatially distributed model	205	47.90
Did the model include future projections? (n = 428)		
Yes	119	27.80
No	309	72.20
Diagnostic method used*		
Validation	264	53.55
Sensitivity	88	17.85
Verification	15	3.04
None of the above	126	25.56
Validation results shown		
Yes	252	95.45
No	7	2.65
In Supplement	5	1.89
Sensitivity results shown		
Yes	77	87.5
No	3	3.41
In Supplement	8	9.09

* = more than one category could be selected within a single publication

Approximately 33% (n = 146) of the models investigated a vector or reservoir (e.g. distribution) rather than a specific disease or pathogen in a vector, reservoir and/or human (considered ‘not applicable’ under the disease or pathogen investigated in Table 2). Of these 146 models, 47% (n = 70) investigated mosquitoes, 18% (n = 26) investigated ticks, 11% (n = 16) investigated sandflies, and 24% (n = 35) investigated another species of vector, or a reservoir. The most commonly investigated diseases included malaria (16%, n = 70), dengue (14%, n = 60), and West Nile fever (7%, n = 31). North America was the most common region modelled (18%, n = 95), followed by Africa (18%, n = 94) and Asia (16%, n = 84), although there was representation from all areas of the world (Table 2). About a third (49%, n = 210) of the models were conducted at a local or regional level (e.g. province or state), 44% (n = 187) were modelling at a country to global level, and the remaining were unspecified (Table 2).

Model outcomes were related to vectors in approximately one half (n = 278) of the models (Table 2), including such outcomes as probability of vector presence or number of infectious vectors in a region. Human-related outcomes (37%, n = 206: number of infectious humans, incidence of disease, prevalence of disease, reproduction number) were the next most common, followed by reservoirs (12%, n = 65: number of infectious reservoirs, prevalence of disease). In models that investigated an importation pathway, about half involved importation via infectious humans (n = 31), followed by importation of a disease via infectious vectors (25%, n = 16), then via a reservoir (22%, n = 14), and finally via the transport of fomites (3%, n = 2).

Most of the publications reported the parameters used in their model in the publication or in supplementary material (88%, n = 377), whereas 12% (n = 51) did not report their parameters (Table 2). A publication had to have provided the parameter names and values to have been considered a publication which reported their parameters, with reasonable certainty that none of the parameters were missed (e.g. if the publication described the impact of climate on model outcomes, it was expected that the model would include parameters related to climate). Climate parameters were included in 75% (n = 322 of 428) of the models, with parameters on temperature and precipitation being the most common climate-related parameters. Parameters related to vectors also were used (58%, n = 248 of 428), these included biting rate, extrinsic incubation period, mortality/longevity, and parameters related to vector development, such as development rate. Parameters related to topography were also used often (54%, n = 230 of 428) including elevation, vegetation, and land use. Just under half of the publications included parameters related to humans (45%, n = 192 of 428) including incubation period, birth/death rate, and duration of infectiousness. Similarly, reservoir parameters (21%, n = 89 of 429) also included incubation period, birth/death rate, and duration of infectiousness. Few models included pathogen parameters (8%, n = 36 of 283) or temperature thresholds for pathogen growth/survival.

Temporal and spatial components were both present in approximately half the models (Table 2). The remaining models included either a temporal or spatial component. The authors projected their model results into the future in 28% (n = 119) of the publications, frequently to the years 2030, 2050, and 2100.

Climate change was investigated in 29% (n = 124) of the mathematical and statistical models, most of which (87%; n = 108) employed climate models (for definition see Table 1) during their investigation to predict the impact of climate change on their outcome of interest (e.g. probability of presence of a vector in a region). The most common climate models used in the mathematical and/or statistical models included were the Hadley Centre for Climate Change (HADCM3), the Commonwealth Scientific and Industrial Research Organization (CSIRO), and the Canadian Centre for Climate Modelling and Analysis (CCCma). Of the publications which used climate models, about half of the publications used the special report on emission scenarios (SRES), and the other half used the representative concentration pathways (RCPs), which replaced SRES in 2014 [28] (for definitions see Table 1).

Model outcomes were evaluated for consistency with the intended outcomes of the model and/or with empirical data in 74% of the publications (n = 367) (Table 2). For publications that included a model validation test, about 95% (n = 252) reported the results of the validation. Similarly, about 88% (n = 77) of the publications that indicated the authors conducted a sensitivity test on their model reported the results of that test.

## Discussion

This scoping review identified and characterized existing predictive and importation models of VBDs in the international literature. A relatively large number of articles presenting VBD models were captured, with a wide range of diseases, modelling approaches, and model outcomes observed among publications.

### Overview of captured publications

Most of the models were published in journal articles. Publications included in this review needed to contain the mathematical and/or statistical model in the publication itself. Therefore, journal articles were more likely to be included because journals typically require a detailed description of any modelling methods included in the publication, whereas in grey literature the model itself may be less likely to be presented. It is also easier to locate indexed publications than non-indexed publications. There has been an increase in publications since 2013 which is likely due to a growing interest in the use of modelling in infectious disease investigations [29, 30], particularly for high profile outbreaks such as the Zika virus introduction into the Americas in 2015 [5, 31–33], and the outbreaks of chikungunya that have occurred globally since 2005 [34]. As well, there have been many advancements in technology, software and computing power that are necessary to run complex mathematical and statistical models [29].

### Models and modelling methods

The most commonly investigated diseases were malaria and dengue. This corroborates previous review publications that have also indicated that malaria and dengue are among the most widely investigated VBDs using mathematical and/or statistical models [9, 17, 35, 36]. This may reflect the high disease burden of malaria and dengue globally [9, 37] and their international importance which has funded research for decades. Thus, there is a large amount of data on these diseases, which is required for accurate model parameterization. Fitting an accurate model for less studied diseases is difficult and often requires substituting parameters from studies done on different diseases or vectors, thus lowering confidence in the model’s predictability. Other globally important diseases may be less studied because there is an effective disease control method available, such as vaccination in the case of yellow fever [38] and Japanese encephalitis [39], versus malaria and dengue, where vaccines are currently being developed and improved [37, 40].

Researchers included a variety of parameters in their models, including parameters related to climate, vectors, humans, reservoirs, and topography. Most of the models included parameters related to climate and topography, both of which are known to impact vector range, and thus the presence of disease, as well as vector and disease range expansion [13, 16]. Not all publications included parameters on the vector, despite all publications investigating VBDs. These publications were focused on transmission in humans and reservoirs [41, 42].

Despite most of the publications reporting the parameters used in their model, 12% of publications did not report their parameters either in the paper itself or in supplemental material. This is concerning, as it affects the reader’s ability to learn from or assess the legitimacy of the model(s) in the publication. With the differences in terminology used, variations in reporting methods between publications, and some of the publications failing to disclose important information relating to their mathematical or statistical models, reporting guidelines would be useful to improve the clarity, consistency, and interpretation for publications that report on mathematical and/or statistical models. There are no widely endorsed guidelines for publishing articles on mathematical and/or statistical models, similar to ones that have been developed for systematic reviews [43] and observational studies [44].

Almost all of the models captured in the scoping review were solely predictive models, and therefore did not investigate the importation of a disease or vector from an endemic region to a non-endemic region. Most of the models that involved importation used mathematical modelling techniques, likely because exploring importation involves simulating scenarios that have yet to occur and statistical modelling is limited to extrapolation of results into the near future. Future work in this area could involve combining importation models with climate models and RCPs/SRES to determine locations where a disease may become an issue under future anticipated climate changes.

### Gaps in knowledge

Climate change is a cause of vector range expansion and thus disease range expansion [9, 45, 46], making climate change an important topic to investigate when conducting mathematical and statistical modeling of VBDs. Modeling allows the researcher to investigate the possible future range expansion of vectors and diseases under different climate change scenarios through the integration of climate models, and climate scenarios, such as RCPs or SRES. This can be used to identify regions where the climate might be suitable for pathogens, vectors, and reservoirs to be jointly present enabling the transmission and spread of diseases to humans [2, 7, 47], allowing for advanced preparation and advocacy. Although many of the models included climate parameters (e.g. current climate data from weather stations), most of the models captured in this scoping review did not explicitly investigate climate change, as they did not use a projected climate model to explore outcomes under projected climate, despite the potential relevance to the spatial and geographic transmission of disease, as well as vector range and distribution. This could be because the focus of most of the mathematical and statistical models captured in this scoping review were on diseases within endemic areas (solely predictive models), rather than disease importations/introductions to new areas (importation models, or models which were both predictive and importation models). With the impacts of climate change becoming increasingly irreversible [48], more investigation of disease distribution, prevalence, and incidence under current and future climate scenarios are valuable for the assessment of future risks.

### Strengths and limitations of the scoping review

This scoping review followed a framework [15], and a protocol was created in part before the start of the review, allowing for a rigorous and reproducible process that minimized review bias. The relevance screening and data charting forms were conducted by two independent reviewers in order to reduce human error and possible biases.

The scoping review inclusion criteria may have introduced some language bias towards English- and French- speaking countries as the search was only conducted in English and only English and French publications were considered for inclusion in the scoping review. For that same reason, there may have been a biased focus on diseases that are more likely to impact English- and French-speaking countries.

By conducting the search using a variety of relevant publication databases, Google for non-indexed and grey literature, and conducting search verification, an attempt was made to minimize the number of relevant publications that were not captured. Despite the steps taken to capture as many relevant publications as possible, some relevant publications may have been missed. Articles were deduplicated through a combination of methods (by author, title, abstract, and journal), making it very likely that the duplicates of articles between databases were found and removed; however, some may have been missed. In addition, due to the wide variety of terminology used in relation to mathematical and statistical models, the key words used may not have captured all of the relevant literature. To augment any sensitivity issues, references lists of included publications were hand screened for relevant publications omitted by the search.

## Conclusion

Researchers creating their own mathematical and/or statistical models on VBDs can use this scoping review to quickly identify published models of the diseases and/or regions of interest to them, and evaluate which parameters have been most useful in those models. These models can be useful in predicting when and where changes in disease distribution may happen, allowing for advanced programming and planning in regions that are more likely to experience an emerging disease or outbreaks of endemic diseases. As globalization and climate change progresses, keeping apprised of the current techniques and approaches that work or do not work is important for infectious disease modellers working in a rapidly changing environment.

## Supporting information

S1 Appendix Scoping review protocol. (DOCX)

S2 Appendix PRISMA-ScR checklist. Preferred Reporting Items for Systematic reviews and Meta-Analyses extension for Scoping Reviews (PRISMA-ScR) Checklist.
(PDF)

S3 Appendix Full list of included publications. (DOCX)

S4 Appendix Data charting of all included publications. (CSV)
