# Supplementary material for: A scoping review of importation and predictive models related to vector-borne diseases, pathogens, reservoirs, or vectors (1999–2016)
Source: PLoS One. 2020 Jan 15;15(1):e0227678. doi: 10.1371/journal.pone.0227678 (PMC6961930; doi:10.1371/journal.pone.0227678)
Supplement: S1 Appendix — (DOCX) [file pone.0227678.s001.docx]

# Title

A scoping review of importation and predictive models for vector-borne diseases, pathogens and/or vectors that exist globally

# Authors

Tara Sadeghieh^1, 2^, Victoria Ng^2^, Lisa A. Waddell^2^, Alexandra Hall^1, 2^, Jan Sargeant^1, 3^

1. Department of Population Medicine, Ontario Veterinary College, University of Guelph, Guelph, Ontario, N1G 2W1, Canada
2. Division of Public Health Risk Sciences, Laboratory for Foodborne Zoonoses, Public Health Agency of Canada, 160 Research Lane, Suite 206, Guelph, Ontario, N1G 5B2, Canada.
3. Centre for Public Health and Zoonoses, Ontario Veterinary College, University of Guelph, Guelph, Ontario, N1G 2W1, Canada

# Important Dates

Protocol initiation – Dec 7, 2016

Initial search – Dec 20, 2016

Contents

[Title 1](#_Toc478980252)

[Authors 1](#_Toc478980253)

[Important Dates 1](#_Toc478980254)

[Background Information 2](#_Toc478980255)

[Definitions 3](#_Toc478980256)

[Objectives of the Scoping Review 3](#_Toc478980257)

[Study Questions 3](#_Toc478980258)

[Study Sub-Questions 3](#_Toc478980259)

[Planned Study Outputs 4](#_Toc478980260)

[Methods 4](#_Toc478980261)

[Review Team Expertise and Responsibilities 4](#_Toc478980262)

[Search Strategy 4](#_Toc478980263)

[Algorithms 4](#_Toc478980264)

[Databases 4](#_Toc478980265)

[Search Verification 4](#_Toc478980266)

[Review Management 4](#_Toc478980267)

[Data Analysis 5](#_Toc478980268)

[Relevance Screening 5](#_Toc478980269)

[Inclusion/Exclusion Criteria 5](#_Toc478980270)

[Data characterization 5](#_Toc478980271)

[Appendix A: Relevance Screening Form 6](#_Toc478980272)

[Appendix B: Data Characterization and Utility (DCU) Tool 8](#_Toc478980273)

[Appendix C: Search Strategy 15](#_Toc478980274)

# Background Information

Vector-borne diseases are diseases which are spread by vectors – arthropods which transmit pathogens between humans, reservoirs and fomites. With globalization and climate change, vector-borne diseases are expected to move outside their usual range. There are several ways that this expansion can occur, including through human travel and transport, animal migration, habitat change, as well as vector range enlargement. Although vector-borne pathogens can enter a non-endemic region, other factors are required for the pathogen to become established in that region, including the existence of local reservoirs, suitable hosts, and endemic vectors which can then transmit the pathogen locally. Climate change is a driving factor in vector and reservoir range expansion. As average temperatures become increasingly warmer and precipitation increases, vectors and reservoirs are expected to move into areas they previously could not due to unfavourable climate and become established.

Mathematical and statistical models can be used to predict the spread of vector-borne diseases, pathogens, and vectors. These models can be useful in informing decisions in public health and medicine, for advocacy and programs surrounding these diseases, and they allow for advance preparation of possible outbreaks. We are interested in predictive models which focus on the distribution or spread of a disease or a component of its transmission cycle, and are used to forecast the temporal and/or geographic spread, and distribution of a disease, pathogen, reservoir or vector, as well as importation models, which are used to predict the introduction and/or establishment and/or movement of a disease/pathogen via a reservoir and/or vector from an endemic region into a non-endemic region. Predictive and importation models exist globally, but it can be difficult to acquire literature involving these models because the terminology used when describing these models is inconsistent.

We are conducting a scoping review to identify and compile predictive and importation models that exist globally, as well as to characterize these models. This review can then be used to inform researchers looking to create models by providing a compilation of model characteristics, such as the variables used in the models. This scoping review will also compile a list of documents which look at models that use Canada and/or the Northern United States as location(s). Models which specifically investigate the impact of climate change on vector-borne diseases, pathogens, and vectors will also be compiled, as climate change is one of the driving factors of vector-borne disease importation and spread. This scoping review will also reveal any gaps that may exist in the literature involving vector-borne predictive and importation models.

Sources:

Ogden, N.H., Milka, R., Caminade, C., and Gachon, P. 2014. Recent and projected future climatic suitability of North America for the Asian tiger mosquito *Aedes albopictus*. Parasites and Vectors 7(532).

Vector-borne diseases. Accessed Dec 16, 2016: http://www.who.int/mediacentre/factsheets/fs387/en/

Wudel, B., and Shadabi, E. [2016. A short review of literature on the effects of climate change on mosquito-borne illnesses in Canada. National Collaborating Centre for Infectious Diseases.](http://www.cmaj.ca/cgi/content/full/161/11/1419)

## Definitions

- **Carrying capacity:** the maximum population size that a region can sustain without environmental degradation
- **Climate change:** Long term changes (lasting more than three decades) in normal climate patterns (including, but not limited to, temperature, precipitation, extreme weather patterns, and sea level changes)
  - **Projected climate:** the estimation of future climate based on the current climate trends
  - **Climate anomalies:** the difference of a future climate compared to the present climate
  - **Global warming:** a gradual increase in the overall temperature of the earth's atmosphere, usually due to the greenhouse effect caused by increased levels of carbon dioxide, and other pollutants.
- **Climate model:** A set of mathematical equations which simulate a climate system
- **Distribution:** describes the areas where the species may exist (normally a map is produced using the model)
- **Fomite:** a non-living object/substance that can harbour and spread pathogens
- **Incidence rate:** number of new cases in humans, reservoirs or vectors over population time at risk during a defined time period
- **Incubation period:** time between exposure to pathogen and symptom onset
- **Importation model:** Mathematical and/or statistical models used to predict the introduction and/or establishment and/or movement of a disease, pathogen, vector and/or reservoir via a reservoir, vector, human, fomite and/or non-reservoir animal from an endemic region into a non-endemic region
- **Mathematical model:** a single or set of equations which simulate or explain a system, and/or forecast future behaviour of that system
  - **Agent based model** (also referred to as microsimulation): simulate the actions and interactions of autonomous agents with their neighbours and their environment based on sets of rules. There may be more than one type of agent. (note: similarly to Stock and flow, agent based can use the state transmission approach). This kind of model may or may not be spatially distributed. Cellular automata show similarities with agent based model but exist within a discrete space (a grid for example)
  - **Discrete event model:** This is a simulation system where the state variable changes according to specific events instead of over regular time steps or continuous time flow (ie. there is no fixed time-step at which point the state of variables are updated). (Note: they are usually used when the element of interest is related to specific event and as such are more often used by engineer to simulate industrial assembly for example).
  - **Ecological niche model:** predicting the distribution of a species in a specified region
  - **Mixed model:** a mix of the previous, combined together to simulate a system. The model may sometimes be referred to as a hybrid model. If choosing this option, there is no need to check off the models that are part of the hybrid
  - **Network model:** A network database consists of a collection of records connected to one another through links. Note: I am pretty confident that if a network model is used, the word “network” will be explicit in the abstract, or even in the title.
  - **Stock and flow:** the dynamic behaviour of the model is driven by flows that accumulate in stocks. (e.g. SI, SIR, SEIR, which are example of state transition models). System dynamics model are usually simulated using a constant time step (dt) and seldom are spatially distributed.
- **Northern USA**: North of the line roughly drawn by the northern borders of Arizona, New Mexico, Oklahoma, Arkansas, Tennessee, and North Carolina
- **Period Prevalence**: proportion of individuals with the disease over a defined time period
- **Predictive model:** Mathematical and/or statistical models used to forecast the temporal and/or geographic spread, and distribution of a disease, pathogen, reservoir or vector.
- **Prevalence**: proportion of individuals with a disease at a certain time period
- **Representative Concentration Pathways Scenario (RCP):** Possible climate futures described via greenhouse gas concentration trajectories. Currently four are used, with RCP2.6 being the least projected rise in greenhouse gas concentrations, and RCP8.5 being the most (**RCP2.6, RCP4.5, RCP6, RCP8.5).**
- **Reproductive number (including basic, effective and type reproduction numbers):** number of cases one case generates on average over the course of its infectious period.
- **Reservoir:** An organism which harbours a pathogen that can multiply without injury/damage to the organism
- **Sensitivity or robustness analysis:** different ways by which “the study of how uncertainty in the output of a model can be attributed to different sources of uncertainty in the model input”. (Saltelli et al 2008); the degree to which the model output changes when changing the input variables (within values dictated by literature and common sense)
- **Socioeconomic information:** information relating to an individual’s level of income, education, and social status
- **Spatial model:** the model is not a “dynamic” model over time and incorporates geographical information (eg. distribution)
- **Statistical methods:** methods of modeling which involve the compilation, analysis and/or interpretation of datasets (eg. regressions)
- **Temporal-spatially distributed model:** A system for which the simulated dependent variable is a function of time and space.
- **Temporal model:** the dependent variable is only a function of time and initial value of state variables.
- **Validation**: determining the degree to which a model is an accurate representation of the real world system the model is simulating
  - **Empirical validation:** the output of the model is assessed against empirical data**.**
  - **Logical validation:** the authors verify that the model behaviour is coherent with the assumptions made or the knowledge of the system (sometimes included in the “verification” step)
- **Vector-borne disease**: Infectious disease, affecting humans, transmitted by an arthropod species, including mosquitoes, ticks, flies, and fleas.
- **Vector-borne pathogen**: A microorganism which can cause disease in humans and is transmitted by vectors
- **Vector**: An arthropod species which aids in the spreading of disease by transmitting pathogens between humans and reservoirs
- **Vector density:** number of mosquitoes per human
- **Verification:** determining whether or not the model output accurately represents the logical framework conceived by the modeller.

# Objectives of the Scoping Review

## Study Questions

1. What **mathematical and/or statistical models** exist globally that predict the **importation** of **vector-borne diseases,** **pathogens, reservoirs and/or vectors** from a non-endemic region to an endemic region (including within and between country/territory importations)?
2. What **mathematical and/or statistical** models exist globally that **forecast** on the **spread, transmission, and distribution** of **vector-borne diseases, pathogens, reservoirs and/or vectors**?

## Study Sub-Questions

1. What are the characteristics of the modelling methods used, including, class of model, explanatory variables used, outcome measures, timeframe, and country/region of interest?
2. What predictive and/or importation models exist that investigate Canada and/or the northern United States as a location?
3. What predictive models exist that investigate the impact of current and projected climate change on vector-borne diseases, pathogens, vectors and reservoirs in Canada and/or the northern United States as a location?

## Planned Study Outputs

1. Description and characteristics of the literature related to predictive and importation models of vector-borne diseases, pathogens, vectors and reservoirs that exist globally.

# Methods

## Review Team Expertise and Responsibilities

The review team has expertise in zoonotic diseases, vector-borne diseases, disease modeling, synthesis research, and library science.

## Search Strategy

### Algorithms

((import or imported or importation or importations or introduction or transmission OR spatial OR spread OR expansion OR expand OR spatiotemporal) AND (model or modeling or modelling or forecast or predict or prediction OR projection or "risk analysis" or "risk assessment" OR "pathway analysis" OR traveller or travellers or traveler or travelers) AND ("vector borne" or vectorborne or vector or vectors or reservoir OR mosquito OR flea OR fly OR tick OR zoonotic OR zoonoses OR zoonosis OR viremic or viraemic or viremia or viraemia or bacteremic or bacteraemic or bacteremia or bacteraemia)) NOT (protein OR transfusion)

The search will be executed with date limits of January 1, 1999 to December 20, 2016 (initial search date).

### Databases

1. MEDLINE (via PubMed)
2. Scopus
3. Web of Science
4. Global Health (via Ovid)
5. GreenFILE (via EBSCOhost)

### Search Verification

Search verification will include searching the reference lists of an initial ten randomly selected relevant papers. If we are no longer identifying potentially relevant papers that were not captured by our search, then reference list screening will be complete. Otherwise the process will continue with additional relevant papers until we no longer identify relevant papers omitted by the search strategy.

Relevant conference abstracts or short papers will be identified and linked to publications or reports by searching through the Distiller database of eligible publications for those from the same authors with a similar title. We will verify whether or not the content of the abstract is presented in the paper before quarantining the abstract as a duplicate citation of the paper. If no publication is identified, we will contact the corresponding author via an email that indicates we are conducting a scoping study, that their research is relevant and of interest to us, and to see if a publication is available. A second email will be sent if no response is received after two weeks. After an additional two weeks the abstract will be considered the only available documentation of the research. If no correspondence information is given, an attempt will be made to find publically available contact information and an email will be sent to one of the authors as described above. If no email is found, no response is given, or no publication is available, we will exclude the abstract or short paper if there is insufficient information for characterization of the research.

# Review Management

The search results will be compiled and deduplicated in EndNote. The results will then be exported to DistillerSR for further deduplication and for management of the scoping review, including relevance screening and data characterization stages.

# Data Analysis

Descriptive statistics and summarization will be conducted in Microsoft Excel and Stata statistical software.

## Relevance Screening

Relevance screening will include the evaluation of title and abstracts to identify citations that may be relevant to the review question. Each citation will be evaluated by two reviewers working independently using the form in Appendix A. Conflicts will be resolved by consensus of the reviewing pair or if necessary by consulting with a third reviewer.

### Inclusion/Exclusion Criteria

- Time Frame: ≥1999 (West Nile cases first recorded in NY in 1999, in Canada in 2001 - http://www.phac-aspc.gc.ca/publicat/ccdr-rmtc/14vol40/dr-rm40-10/dr-rm40-10-comm-eng.php)
- Language: English, French
- Country: All
- Document Type: journal articles, theses, grey-literature; excluding reviews, editorials and commentaries

# Data characterization

The goal of data characterization is to confirm that each full paper is relevant and then characterize pertinent information required to appropriately describe each predictive or importation model using the data characterization and utility form in Appendix B. Each full paper will be evaluated by two reviewers working independently. Conflicts will be resolved by consensus of the reviewing pair or if necessary by consulting with a third reviewer.

# Appendix A: Relevance Screening Form

| **Question** | **Options** | **Definitions/additional notes** |
| --- | --- | --- |
| 1. Does the abstract describe an importation and/or predictive model on the distribution or spread of a disease or component of its transmission cycle? | - Yes - No (exclude) - Not clear | **Predictive model on the distribution or spread of a disease or component of its transmission cycle:** Mathematical and/or statistical models used to forecast the temporal and/or geographic spread, and distribution of a disease, pathogen, reservoir or vector.  **Importation model:** Mathematical and/or statistical models used to predict the introduction and/or establishment and/or movement of a disease/pathogen via a reservoir and/or vector from an endemic region into a non-endemic region |
| 1. Is the model forecasting or predicting the importation, spread, distribution, or transmission of a vector-borne disease, pathogen, vector, or reservoir associated with a vector-borne disease that affects humans? | - Yes - No (exclude) | **Vector-borne disease**: Infectious disease, affecting humans, transmitted by an arthropod species, including mosquitoes, ticks, flies, and fleas.  **Vector-borne pathogen**: A microorganism which can cause disease in humans and is transmitted by vectors  **Vector**: An arthropod species which aids in the spreading of disease by transmitting pathogens between humans and reservoirs  **Reservoir:** An organism which harbours a pathogen that can multiply without injury/damage to the organism |
| 1. The model is described in what type of document? | - Journal article - Thesis/dissertation - Conference proceeding paper or abstract - Report from government or another source - Other grey literature - Not clear - None of the above (exclude) | **Journal article**: research/investigation/study carried out by the researcher (incl. surveys, interviews, outbreak reports, observations, etc.) and published in a journal. Only include reviews if the abstract describes model development and/or use, and the author(s) of the review were the one(s) who developed the model or used the model in a way unique to its original developer(s). Otherwise, it is considered “none of the above”.  **Thesis/dissertation**: A long paper/essay involving personal research (usually written for a university degree)  **Grey Literature:** Research that is unpublished or published in a non-commercial form, including, but not limited to **conference proceeding abstracts** (short synopsis of a research project presented at a conference) and **reports** from governments or other sources. The grey literature must describe model development and/or use, and the author(s) of the grey literature must be the one(s) who developed the model or used the model in a way unique to its original developer(s). Otherwise, it is considered “none of the above”. |

# Appendix B: Data Characterization and Utility (DCU) Tool

| **Question** | **Options** | **Definitions/additional notes** |
| --- | --- | --- |
| 1. Is the article published in English or French? | - English - French - Other, please specify: ____ (exclude) |  |
| 1. Does this article focus on vector-borne diseases, pathogens, reservoirs, and/or vectors associated with a vector-borne disease that affects humans? | - Yes - No (exclude) | **Vector-borne disease**: Infectious disease, affecting humans, transmitted by an arthropod species, including mosquitoes, ticks, flies, and fleas.  **Vector-borne pathogen**: A microorganism which can cause disease in humans and is transmitted by vectors  **Vector**: An arthropod species which aids in the spreading of disease by transmitting pathogens between humans and reservoirs  **Reservoir:** An organism which harbours a pathogen that can multiply without injury/damage to the organism |
| 1. What type of model is described in the publication? | - Importation - Predictive - Both importation and predictive - Other (exclude) | **Predictive model on the distribution or spread of a disease or component of its transmission cycle:** Mathematical and/or statistical models used to forecast the temporal and/or geographic spread, and distribution of a disease, pathogen, reservoir or vector.  **Importation model:** Mathematical and/or statistical models used to predict the introduction and/or establishment and/or movement of a disease, pathogen, vector and/or reservoir via a reservoir, vector, human, fomite and/or non-reservoir animal from an endemic region into a non-endemic region |
| 1. The model is described in what type of document? | - Journal article - Thesis/dissertation - Conference proceeding paper or abstract - Report from government or another source - Other grey literature - Not clear - None of the above (exclude) | **Journal article**: research/investigation/study carried out by the researcher (incl. surveys, interviews, outbreak reports, observations, etc.) and published in a journal. Only include reviews if the abstract describes model development and/or use, and the author(s) of the review were the one(s) who developed the model or used the model in a way unique to its original developer(s). Otherwise, it is considered “none of the above”. Does not include editorials or commentaries.  **Thesis/dissertation**: A long paper/essay involving personal research (usually written for a university degree)  **Grey Literature:** Research that is unpublished or published in a non-commercial form, including, but not limited to **conference proceeding abstracts** (short synopsis of a research project presented at a conference) and **reports** from governments or other sources. The grey literature must describe model development and/or use, and the author(s) of the grey literature must be the one(s) who developed the model or used the model in a way unique to its original developer(s). Otherwise, it is considered “none of the above”. |
| **If an exclusion criteria was chosen above, please submit** | | |
| 1. What vector borne disease/pathogen has the model been developed for?   (Check all that apply) | - Chikungunya - Dengue fever - Rift Valley fever - Yellow fever - Zika - Malaria - Japanese encephalitis - West Nile fever - Leishmaniasis - Crimean-Congo haemorrhagic fever - Lyme disease - Chagas disease/American trypanosomiasis - Sleeping sickness/African trypanosomiasis - Plague - Schistosomiasis - Other, please specify: ____ - Not specified - Not applicable (eg. the model was on the vector) |  |
| 1. Did the model investigate the impacts of climate change? | - Yes - No | **Climate change:** Long term changes (lasting more than three decades) in normal climate patterns (including, but not limited to, temperature, precipitation, extreme weather patterns, and sea level changes)  **Projected climate:** the estimation of future climate based on the current climate trends  **Climate anomalies:** the difference of a future climate compared to the present climate  **Global warming:** a gradual increase in the overall temperature of the earth's atmosphere, usually due to the greenhouse effect caused by increased levels of carbon dioxide, and other pollutants. |
| 1. Which regions were modelled?   (check all that apply) | - North America   - Canada   - Northern USA   - Southern USA   - Mexico - Central America/South America/Caribbean - Africa (includes India Ocean Islands) - Europe (except Russia) - Russia - Asia (includes Middle East, Turkey, Japan) - Australasia and New Zealand - Oceania (includes Fiji, Micronesia, Polynesia and other Pacific island countries) - Global - Not reported/specified | In the case of international importation and/or spread please check off the region of origin and importation  Check off global if the predictions were made for all regions/world wide  **Northern USA**: North of the line roughly drawn by the northern borders of Arizona, New Mexico, Oklahoma, Arkansas, Tennessee, and North Carolina  Russia is indicated individually because of the difficulty to relate conditions to only one continent (part of Russia might be more closely related to Europe while other more to Asia) |
| 1. What was the scale of the model? | - Global - Multi-country - Country - Regional - Local - Unspecified | **Regional:** includes areas below country-level scale, but above local-level, and includes, but is not limited to provinces, states, and counties.  **Local:** at or below the scale of a town, city, village, etc |
| 1. Please record the current and projected years that the model is based on. | - Discrete, please specify: _____ - Interval, please specify: ____ - Not applicable (ie: there are no future predictions, the model is based on current data.) | Please use discrete when there are single (eg. 2050) or multiple years (eg. 2040 and 2080), and use interval when the projection is for a span of years (eg. 2040 to 2080).  Include present years if provided  Indicate NR in the discrete option if the model projected future outcomes, but no year was provided. |
| 1. If the model involved importation, what was the importation pathway?   (check all that apply) | - Vector - Human - Reservoir - Animal (not reservoir) - Fomite - Other, please specify: ________ - N/A not an importation model | **Vector:** An arthropod species which aids in the spreading of disease by transmitting pathogens from host to host  **Reservoir:** An organism which harbours a pathogen that can multiply without injury/damage to the organism  **Fomite:** a non-living object/substance that can harbour and spread pathogens |
| 1. Which of the following were included in the model output? Please specify the outcome that was modelled.   (Check all that apply) | - Vector, please specify: _______   - Number of vectors   - Incidence rate, incidence density   - Incidence risk, incidence proportion, cumulative incidence   - Prevalence or period prevalence   - Probability of presence in a region   - Reproduction number (R0,Reff,RT)   - Distribution   - Other, please specify: ________ - Human, please specify if the model is about a particular subset of the population (e.g. military, healthcare workers, pregnant women, and immunocompromised individuals) _______   - Number of humans   - Incidence rate, incidence density   - Incidence risk, incidence proportion, cumulative incidence   - Prevalence or period prevalence   - Probability of presence in a region   - Reproduction number (R0,Reff,RT)   - Distribution   - Other, please specify: ________ - Reservoir, please specify: ______   - Number of reservoirs   - Incidence rate, incidence density   - Incidence risk, incidence proportion, cumulative incidence   - Prevalence or period prevalence   - Probability of presence in a region   - Reproduction number (R0,Reff,RT)   - Distribution   - Other, please specify: ________ - Fomite, please specify: _______   - Number of fomites   - Probability of presence in a region   - Reproduction number (R0,Reff,RT)   - Distribution   - Other, please specify: ________ - Other, please specify: _________   - Incidence rate, incidence density   - Incidence risk, incidence proportion, cumulative incidence   - Prevalence or period prevalence   - Probability of presence in a region   - Reproduction number (R0,Reff,RT)   - Distribution   - Other, please specify: ________ | In the accompanying text boxes please include the variable name as specified by the author, or other important information such as the specific species.  **Vector:** An arthropod species which aids in the spreading of disease by transmitting pathogens from host to host  **Vector-borne pathogen**: A microorganism which can cause disease in humans and is transmitted by vectors  **Reservoir:** An organism which harbours a pathogen that can multiply without injury/damage to the organism  **Fomite:** a non-living object/substance that can harbour and spread pathogens  **Incidence rate:** number of new cases in humans, reservoirs or vectors over population time at risk during a defined time period  **Prevalence**: proportion of individuals with a disease at a certain time period  **Period Prevalence**: proportion of individuals with the disease over a defined time period  **Reproductive number (including basic, effective and type reproduction numbers):** number of cases one case generates on average over the course of its infectious period.  **Distribution:** describes the areas where the species may exist (normally a map is produced using the model) |
| 1. What parameters required **external data** to be used in the model?   (Check all that apply) | - Climate   - Temperature   - Precipitation   - Humidity   - Seasonality   - Cloud cover   - Wind speed   - Solar radiation   - Growing degree days   - Snow cover   - Isothermality   - Photoperiod   - Pressure   - Not specified   - (dynamic list) - Vector (invading or endemic), specify species: _______   - Distribution   - Related to movement/spread   - Population size   - Density of vector per human   - Bite rate   - Mortality/longevity   - Related to life stages (eg. number of larvae)   - Carrying capacity   - Transmission probability of pathogen per bite (vector to human or reservoir)   - Extrinsic incubation period   - Not specified   - (dynamic list) - Human, please specify if the model is about a particular subset of the population (e.g. military, healthcare workers, pregnant women, and immunocompromised individuals) _______   - Socioeconomic information   - Mobility/travel between regions   - Mobility/travel within regions   - Birth/death rate   - Population size   - Contact rate   - Recovery rate   - Distribution   - Incubation period   - Transmission probability of pathogen per bite (human to vector)   - Not specified   - Other (dynamic list) - Topography   - Land use   - Vegetation   - Elevation   - Slope   - Building density   - Not specified   - Other (dynamic list) - Reservoir, specify species: ______   - Mobility/travel between regions   - Mobility/travel within regions   - Birth/death rate   - Population size   - Contact rate   - Incubation period   - Transmission probability of pathogen per bite (Reservoir to vector)   - Density   - Distribution   - Not specified   - Other (dynamic list) - Fomite (dynamic list), please specify: __________ - Pathogen (dynamic list), specify species: __________ - Not specified - Other, please specify: _________ - Not reported | In the accompanying text boxes please include the **variable name** as specified by the author, or other important information such as annual vs. seasonal temperature and/or the **specific species**. Include information on whether or not all the variables were reported, if possible (eg. the author stated they used eight variables, but only reported three).  Do not include baseline parameters (eg. using vector distribution during the present as the baseline, which then becomes the outcome for the prediction).  **Vector density:** number of mosquitoes per human  **Socioeconomic information:** information relating to an individual’s level of income, education, and social status  **Incubation period:** time between exposure to pathogen and symptom onset  **Carrying capacity:** the maximum population size that a region can sustain without environmental degradation |
| 1. Were all the variables in the model reported on in the paper? | - Yes - No |  |
| 1. Was a climate model used/integrated with the model? | - Yes, please specify which climate model and Representative Concentration Pathway Scenario were used: ________ - No | **Climate model:** A set of mathematical equations which simulate a climate system  **Representative Concentration Pathways Scenario (RCP):** Possible climate futures described via greenhouse gas concentration trajectories. Currently four are used, with RCP2.6 being the least projected rise in greenhouse gas concentrations, and RCP8.5 being the most (**RCP2.6, RCP4.5, RCP6, RCP8.5).**  Previously, Special Report on Emissions Scenarios (SRES) were used: A1(A1FI, A1B, A1T), A2, B1, B2 |
| 1. Please indicate whether a mathematical and/or statistical model was used and specify the model name according to the authors.   (Check all that apply) | - Mathematical models, please specify: _________ - Statistical methods, please specify: _____ - Other, specify: ________ | In the accompanying text boxes please include the name of model as specified by the author  **Mathematical model:** a single or set of equations which simulate or explain a system, and/or forecast future behaviour of that system  **Statistical methods:** methods of modeling which involve the compilation, analysis and/or interpretation of datasets (eg. regressions)  Possible mathematical models:  **Stock and flow:** the dynamic behaviour of the model is driven by flows that accumulate in stocks. (e.g. SI, SIR, SEIR, which are example of state transition models). System dynamics model are usually simulated using a constant time step (dt) and seldom are spatially distributed.  **Agent based model** (also referred to as microsimulation): simulate the actions and interactions of autonomous agents with their neighbours and their environment based on sets of rules. There may be more than one type of agent. (note: similarly to Stock and flow, agent based can use the state transmission approach). This kind of model may or may not be spatially distributed. Cellular automata show similarities with agent based model but exist within a discrete space (a grid for example)  **Discrete event model:** This is a simulation system where the state variable changes according to specific events instead of over regular time steps or continuous time flow (ie. there is no fixed time-step at which point the state of variables are updated). (Note: they are usually used when the element of interest is related to specific event and as such are more often used by engineer to simulate industrial assembly for example).  **Network model:** A network database consists of a collection of records connected to one another through links. Note: I am pretty confident that if a network model is used, the word “network” will be explicit in the abstract, or even in the title.  **Mixed model:** a mix of the previous, combined together to simulate a system. The model may sometimes be referred to as a hybrid model. If choosing this option, there is no need to check off the models that are part of the hybrid  **Ecological niche model:** predicting the distribution of a species in a specified region |
| 1. Please indicate whether or not the model incorporates time and space | - Temporal-spatially distributed model - Temporal only - Spatial only - Neither temporal nor spatial | **Temporal-spatially distributed model:** A system for which the simulated dependent variable is a function of time and space.  **Temporal model:** the dependent variable is only a function of time and initial value of state variables.  **Spatial model:** the model is not a “dynamic” model over time and incorporates geographical information (eg. distribution) |
| 1. Indicate whether the verification, calibration, validation and sensitivity analysis results were performed?   (check all that apply) | - Verification performed - Validation performed - Empirical validation - Explicit logical validation - Sensitivity analysis performed - None of the above | **Verification:** determining whether or not the model output accurately represents the logical framework conceived by the modeller.  **Validation**: determining the degree to which a model is an accurate representation of the real world system the model is simulating   - **Empirical validation:** the output of the model is assessed against empirical data**.** - **Logical validation:** the authors verify that the model behaviour is coherent with the assumptions made or the knowledge of the system (sometimes included in the “verification” step)   **Sensitivity or robustness analysis:** different ways by which “the study of how uncertainty in the output of a model can be attributed to different sources of uncertainty in the model input”. (Saltelli et al 2008); the degree to which the model output changes when changing the input variables (within values dictated by literature and common sense) |
| 1. Are the results of the validation test presented?   (if validation was checked for Q14) | - Yes - Yes, in the supplementary material - No |  |
| 1. Are the results of the sensitivity analysis presented?   (if sensitivity analysis was checked for Q14) | - Yes - Yes, in the supplementary material - No |  |
| 1. What program was used to execute the model? | Text | If not reported put NR |
| 1. Other comments | Text | Add notes on details you think are important to the project and not captured in the questions above, including statements on the limitations or recommendations on the methods and/or models used. |

# Appendix C: Search Strategy

((import or imported or importation or importations or introduction or transmission OR spatial OR spread OR expansion OR expand OR spatiotemporal) AND (model or modeling or modelling or forecast or predict or prediction OR projection or "risk analysis" or "risk assessment" OR "pathway analysis" OR traveller or travellers or traveler or travelers) AND ("vector borne" or vectorborne or vector or vectors or reservoir OR mosquito OR flea OR fly OR tick OR zoonotic OR zoonosis OR zoonoses OR viremic or viraemic or viremia or viraemia or bacteremic or bacteraemic or bacteremia or bacteraemia)) NOT (protein OR transfusion)

Limited 1999 - present

Dec 20, 2016

PubMed – 7688 (all fields)

Scopus – 40527 (title, abs, key) – too many hits; limited some search terms to title only

Web of Science – 23416 (topic) – too many hits; limited some search terms to title only

Ovid (Global Health) – 5750 (all fields)

EBSCOHost (GreenFILE) – 873 (all fields)

(TITLE-ABS-KEY(import OR imported OR importation OR importations OR spread OR spatiotemporal) OR TITLE(introduction OR transmission OR spatial OR expand OR expansion)) AND TITLE-ABS-KEY(model OR modeling OR modelling OR forecast OR predict OR prediction OR projection OR "risk analysis" OR "risk assessment" OR "pathway analysis" OR traveller OR traveler OR travellers OR travelers) AND (TITLE-ABS-KEY("vector borne" OR vectorborne OR vector OR vectors OR mosquito OR flea OR fly OR tick OR zoonotic OR zoonoses OR zoonosis OR viremic OR viraemic OR viremia OR viraemia OR bacteremic OR bacteraemic OR bacteremia OR bacteraemia) OR TITLE(reservoir)) AND NOT (protein) AND NOT (transfusion) AND PUBYEAR > 1998

Dec 20, 2016

Web of Science – 6073 (topic, except: title for introduction, transmission, spatial, expand, expansion and reservoir)

Dec 22, 2016

Scopus – 9201 (title, abs, key except: title for introduction, transmission, spatial, expand, expansion and reservoir)

Total before deduplication: 29585

Total after EndNote deduplication: 19864

Total after Distiller deduplication: 19710

Total after relevance screening: 511
