# Supplementary material for: A scoping review of importation and predictive models related to vector-borne diseases, pathogens, reservoirs, or vectors (1999–2016)
Source: PLoS One. 2020 Jan 15;15(1):e0227678. doi: 10.1371/journal.pone.0227678 (PMC6961930; doi:10.1371/journal.pone.0227678)
Supplement: S3 Appendix — (DOCX) [file pone.0227678.s003.docx]

1. Abdullahi MB, Abu Hasan Y, Abdullah FA. Optimal strategy for controlling the spread of *Plasmodium knowlesi* malaria: Treatment and culling. International Conference on Mathematics, Engineering and Industrial Applications 2014. 2015;1660.

2. Abeyrathna MPAR, Abeygunawrdane DA, Wijesundara RAAV, Mudalige VB, Bandara M, Perera S, et al. Dengue propagation prediction using human mobility. 2nd International Moratuwa Engineering Research Conference, MERCon 2016. 2016:156-61.

3. Addawe J, Pajimola AK. Dynamics of climate-based malaria transmission model with age-structured human population. AIP Conference Proceedings. 2016;1782.

4. Adde A, Roux E, Mangeas M, Dessay N, Nacher M, Dusfour I, et al. Dynamical mapping of *Anopheles darlingi* densities in a residual malaria transmission area of French Guiana by using remote sensing and meteorological data. Plos One. 2016;11:20.

5. Agusto FB, Gumel AB, Parham PE. Qualitative assessment of the role of temperature variations on malaria transmission dynamics. Journal of Biological Systems. 2015;23:597-630.

6. Agusto FB, Marcus N, Okosun KO. Application of optimal control to the epidemiology of malaria. Electron J Differ Equ. 2012:22.

7. Albert M, Wardrop NA, Atkinson PM, Torr SJ, Welburn SC. Tsetse fly (*G. f. fuscipes*) distribution in the Lake Victoria basin of Uganda. PLoS neglected tropical diseases. 2015;9:e0003705.

8. Althouse BM, Vasilakis N, Sall AA, Diallo M, Weaver SC, Hanley KA. Potential for Zika virus to establish a sylvatic transmission cycle in the Americas. PLoS neglected tropical diseases. 2016;10:e0005055.

9. Altobelli A, Boemo B, Mignozzi K, Bandi M, Floris R, Menardt G, et al. Spatial Lyme borreliosis risk assessment in north-eastern Italy. International Journal of Medical Microbiology. 2008;298:125-8.

10. Amek N, Bayoh N, Hamel M, Lindblade KA, Gimnig JE, Odhiambo F, et al. Spatial and temporal dynamics of malaria transmission in rural Western Kenya. Parasites Vectors. 2012;5:13.

11. Arsevska E, Hellal J, Mejri S, Hammami S, Marianneau P, Calavas D, et al. Identifying areas suitable for the occurrence of Rift Valley fever in North Africa: Implications for surveillance. Transboundary and Emerging Diseases. 2016;63:658-74.

12. Attaway DF, Jacobsen KH, Falconer A, Manca G, Waters NM. Risk analysis for dengue suitability in Africa using the ArcGIS predictive analysis tools (PA tools). Acta Trop. 2016;158:248-57.

13. Ayala D, Costantini C, Ose K, Kamdem GC, Antonio-Nkondjio C, Agbor JP, et al. Habitat suitability and ecological niche profile of major malaria vectors in Cameroon. Malaria Journal. 2009;8:307.

14. Ayyaz A, Muaz U, Awan SM, Ayyaz MN. Simulation model for counter-measures against *Aedes aegypti*. Proceedings - 2015 13th International Conference on Frontiers of Information Technology, FIT 2015. 2015:98-103.

15. Baeza A, Bouma MJ, Dhiman R, Pascual M. Malaria control under unstable dynamics: Reactive vs. climate-based strategies. Acta Trop. 2013;129:42-51.

16. Banu S, Hu W, Guo Y, Hurst C, Tong S. Projecting the impact of climate change on dengue transmission in Dhaka, Bangladesh. Environment international. 2013;63:137-42.

17. Barbazan P, Guiserix M, Boonyuan W, Tuntaprasart W, Pontier D, Gonzalez JP. Modelling the effect of temperature on transmission of dengue. Medical and Veterinary Entomology. 2010;24:66-73.

18. Barbu C, Dumonteil E, Gourbiere S. Optimization of control strategies for non-domiciliated *Triatoma dimidiata*, Chagas disease vector in the Yucatan Peninsula, Mexico. PLoS neglected tropical diseases. 2009;3:e416.

19. Barrios J, Piétrus A, Joya G, Marrero A, Arazoza H. A differential inclusion approach for modeling and analysis of dynamical systems under uncertainty: Application to dengue disease transmission. Soft Computing. 2013;17:239-53.

20. Barrios JM, Verstraeten WW, Maes P, Aerts JM, Farifteh J, Coppin P. Using the gravity model to estimate the spatial spread of vector-borne diseases. International Journal of Environmental Research and Public Health. 2012;9:4346-64.

21. Bee TK, Lye KH, Yean TS. Modeling dengue fever subject to temperature change. 6th International Conference on Fuzzy Systems and Knowledge Discovery, FSKD 2009. 2009;5:61-5.

22. Benedict MQ, Levine RS, Hawley WA, Lounibos LP. Spread of the tiger: Global risk of invasion by the mosquito *Aedes albopictus*. Vector borne and zoonotic diseases (Larchmont, NY). 2007;7:76-85.

23. Bergsman LD, Hyman JM, Manore CA. A mathematical model for the spread of West Nile virus in migratory and resident birds. Mathematical Biosciences and Engineering. 2016;13:401-24.

24. Bietolini S, Candura F, Coluzzi M. Spatial and long term temporal distribution of the *Anopheles maculipennis* complex species in Italy. Special Issue: Gaia Luoni 1970-2004. 2006;48:581-608.

25. Bisanzio D, Giacobini M, Bertolotti L, Mosca A, Balbo L, Kitron U, et al. Spatio-temporal patterns of distribution of West Nile virus vectors in eastern Piedmont Region, Italy. Parasites Vectors. 2011;4:11.

26. Boeckmann M, Joyner TA. Old health risks in new places? An ecological niche model for *I. ricinus* tick distribution in Europe under a changing climate. Health & place. 2014;30:70-7.

27. Bogoch, II, Brady OJ, Kraemer MU, German M, Creatore MI, Brent S, et al. Potential for Zika virus introduction and transmission in resource-limited countries in Africa and the Asia-Pacific region: A modelling study. The Lancet Infectious diseases. 2016;16:1237-45.

28. Bomblies A, Eltahir EA. Assessment of the impact of climate shifts on malaria transmission in the Sahel. Ecohealth. 2010;6:426-37.

29. Bosch J, Muñoz MJ, Martínez M, e la Torre A, Estrada-Peña A. Vector-borne pathogen spread through ticks on migratory birds: A probabilistic spatial risk model for South-Western Europe. Transboundary and Emerging Diseases. 2013;60:403-15S.

30. Bouden M, Moulin B, Gosselin P. The geosimulation of West Nile virus propagation: A multi-agent and climate sensitive tool for risk management in public health. International journal of health geographics. 2008;7:35.

31. Bouden M, Moulin B, Gosselin P. Epidemic propagation of West Nile virus using a multi-agent geo-simulation under various short-term climate scenarios. Proceedings of the 2008 Spring Simulation Multiconference, SpringSim'08. 2008:98-105.

32. Bouden M, Moulin B, Gosselin P. A geosimulation tool to assess intervention scenarios in relation to the spread of infectious disease and environmental factors. Modelling for Environment's Sake: Proceedings of the 5th Biennial Conference of the International Environmental Modelling and Software Society, iEMSs 2010. 2010;3:2257-64.

33. Bouzid M, Colón-González FJ, Lung T, Lake IR, Hunter PR. Climate change and the emergence of vector-borne diseases in Europe: Case study of dengue fever. BMC Public Health. 2014;14.

34. Brady OJ, Golding N, Pigott DM, Kraemer MU, Messina JP, Reiner RC, et al. Global temperature constraints on *Aedes aegypti* and *Ae. albopictus* persistence and competence for dengue virus transmission. Parasites & vectors. 2014;7:338.

35. Brown HE, Yates KF, Dietrich G, MacMillan K, Graham CB, Reese SM, et al. An acarologic survey and *Amblyomma americanum* distribution map with implications for tularemia risk in Missouri. Am J Trop Med Hyg. 2011;84:411-9.

36. Brownstein JS, Holford TR, Fish D. A climate-based model predicts the spatial distribution of the Lyme disease vector *Ixodes scapularis* in the United States. Environmental health perspectives. 2003;111:1152-7.

37. Brownstein JS, Holford TR, Fish D. Effect of climate change on Lyme disease risk in North America. EcoHealth. 2005;2:38-46.

38. Brownstein JS, Rosen H, Purdy D, Miller JR, Merlino M, Mostashari F, et al. Spatial analysis of West Nile virus: Rapid risk assessment of an introduced vector-borne zoonosis. Vector borne and zoonotic diseases (Larchmont, NY). 2003;2:157-64.

39. Brugger K, Rubel F. Simulation of climate-change scenarios to explain Usutu-virus dynamics in Austria. 2009;88:24-31.

40. Buhnerkempe MG, Eisen RJ, Goodell B, Gage KL, Antolin MF, Webb CT. Transmission shifts underlie variability in population responses to *Yersinia pestis* infection. Plos One. 2011;6:8.

41. Butterworth MK, Morin CW, Comrie AC. An analysis of the potential impact of climate change on dengue transmission in the Southeastern United States. Environmental health perspectives. 2016.

42. Caminade C, Ndione JA, Kebe CMF, Jones AE, Danuor S, Tay S, et al. Mapping Rift Valley fever and malaria risk over West Africa using climatic indicators. Atmospheric Science Letters. 2011;12:96-103.

43. Capinha C, Rocha J, Sousa C. Macroclimate determines the global range limit of *Aedes aegypti*. EcoHealth. 2014;11:420-8.

44. Carbajo AE, Vezzani D. Waiting for chikungunya fever in Argentina: Spatio-temporal risk maps. Memorias do Instituto Oswaldo Cruz. 2015;110:259-62.

45. Cardoso-Leite R, Vilarinho AC, Novaes MC, Tonetto AF, Vilardi GC, Guillermo-Ferreira R. Recent and future environmental suitability to dengue fever in Brazil using species distribution model. Transactions of the Royal Society of Tropical Medicine and Hygiene. 2014;108:99-104.

46. Carlson CJ, Dougherty ER, Getz W. An ecological assessment of the pandemic threat of Zika virus. Plos Neglected Tropical Diseases. 2016;10:18.

47. Carolan K, Ebong SM, Garchitorena A, Landier J, Sanhueza D, Texier G, et al. Ecological niche modelling of Hemipteran insects in Cameroon; the paradox of a vector-borne transmission for *Mycobacterium ulcerans*, the causative agent of Buruli ulcer. International journal of health geographics. 2014;13:44.

48. Carvalho BM, Rangel EF, Ready PD, Vale MM. Ecological niche modelling predicts southward expansion of *Lutzomyia (nyssomyia) flaviscutellata (Diptera: Psychodidae: Phlebotominae)*, vector of *Leishmania (leishmania) amazonensis* in South America, under climate change. 2015;10:e0143282.

49. Cauchemez S, Ledrans M, Poletto C, Quenel P, e Valk H, Colizza V, et al. Local and regional spread of chikungunya fever in the Americas. Eurosurveillance. 2014;19.

50. Ceccarelli S, Rabinovich JE. Global climate change effects on Venezuela's vulnerability to Chagas disease is linked to the geographic distribution of five triatomine species. Journal of medical entomology. 2015;52:1333-43.

51. Chalghaf B, Chlif S, Mayala B, Ghawar W, Bettaieb J, Harrabi M, et al. Ecological niche modeling for the prediction of the geographic distribution of cutaneous leishmaniasis in Tunisia. The American journal of tropical medicine and hygiene. 2016;94:844-51.

52. Chamchod F, Cosner C, Cantrell RS, Beier JC, Ruan SG. Transmission dynamics of Rift Valley fever virus: Effects of live and killed vaccines on epizootic outbreaks and enzootic maintenance. Front Microbiol. 2016;6:15.

53. Chao DL, Longini IM, Halloran ME. The effects of vector movement and distribution in a mathematical model of dengue transmission. Plos One. 2013;8:6.

54. Chen CC, Epp T, Jenkins E, Waldner C, Curry PS, Soos C. Predicting weekly variation of *Culex tarsalis (Diptera: Culicidae)* West Nile virus infection in a newly endemic region, the Canadian prairies. J Med Entomol. 2012;49:1144-53.

55. Chen CC, Jenkins E, Epp T, Waldner C, Curry PS, Soos C. Climate change and West Nile virus in a highly endemic region of North America. International journal of environmental research and public health. 2013;10:3052-71.

56. Chen S, Blanford JI, Fleischer SJ, Hutchinson M, Saunders MC, Thomas MB. Estimating West Nile virus transmission period in Pennsylvania using an optimized degree-day model. 2013;13:489-97.

57. Chien L-C, Yu H-L. Impact of meteorological factors on the spatiotemporal patterns of dengue fever incidence. Environment International. 2014;73:46-56.

58. Chuang TW, Wimberly MC. Remote sensing of climatic anomalies and West Nile virus incidence in the northern Great Plains of the United States. PLoS One. 2012;7:e46882.

59. Cianci D, Hartemink N, Ibáñez-Justicia A. Modelling the potential spatial distribution of mosquito species using three different techniques. International Journal of Health Geographics. 2015;14.

60. Cisse B, El Yacoubi S, Gourbiere S. A cellular automaton model for the transmission of Chagas disease in heterogeneous landscape and host community. Appl Math Model. 2016;40:782-94.

61. Clapham H, Moore S, Mills HL, Salje H, Collins C, Rodriquez-Barraquer I, et al. Potential effect of population and climate changes on global distribution of dengue fever: An empirical model. Proceedings of the National Academy of Sciences of the United States of America. 2016;360:830-4.

62. Cleckner H, Allen TR. Dasymetric mapping and spatial modeling of mosquito vector exposure, Chesapeake, Virginia, USA. ISPRS International Journal of Geo-Information. 2014;3:891-913.

63. Cleckner HL, Allen TR, Bellows AS. Remote sensing and modeling of mosquito abundance and habitats in coastal Virginia, USA. 2011;3:2663-81.

64. Click Lambert R, Kolivras KN, Resler LM, Brewster CC, Paulson SL. The potential for emergence of Chagas disease in the United States. Geospatial health. 2008;2:227-39.

65. Conley AK, Fuller DO, Haddad N, Hassan AN, Gad AM, Beier JC. Modeling the distribution of the West Nile and Rift Valley Fever vector *Culex pipiens* in arid and semi-arid regions of the Middle East and North Africa. Parasites & vectors. 2014;7:289.

66. Conte A, Candeloro L, Ippoliti C, Monaco F, De Massis F, Bruno R, et al. Spatio-temporal identification of areas suitable for West Nile disease in the Mediterranean Basin and Central Europe. Plos One. 2015;10:16.

67. Cordovez JM, Rendon LM, Gonzalez C, Guhl F. Using the basic reproduction number to assess the effects of climate change in the risk of Chagas disease transmission in Colombia. Acta Trop. 2014;129:74-82.

68. Costa J, Dornak LL, Almeida CE, Peterson AT. Distributional potential of the *Triatoma brasiliensis* species complex at present and under scenarios of future climate conditions. Parasites & vectors. 2014;7:238.

69. Coudeville L, Baurin N, L'Azou M, Guy B. Potential impact of dengue vaccination: Insights from two large-scale phase III trials with a tetravalent dengue vaccine. Vaccine. 2016;34:6426-35.

70. Coudeville L, Garnett GP. Transmission dynamics of the four dengue serotypes in Southern Vietnam and the potential impact of vaccination. Plos One. 2012;7:11.

71. Cuervo PF, Fantozzi MC, Di Cataldo S, Cringoli G, Mera y Sierra R, Rinaldi L. Analysis of climate and extrinsic incubation of *Dirofilaria immitis* in southern South America. Geospatial Health. 2013;8:175-81.

72. Cunze S, Koch LK, Kochmann J, Klimpel S. *Aedes albopictus* and *Aedes japonicus* - two invasive mosquito species with different temperature niches in Europe. Parasites Vectors. 2016;9:12.

73. Cunze S, Kochmann J, Koch LK, Klimpel S. *Aedes albopictus* and its environmental limits in Europe. PLoS One. 2016;11:e0162116.

74. Deng C, Tao H, Ye Y. Agent-based modeling to simulate the dengue spread. Proceedings of SPIE - The International Society for Optical Engineering. 2008;7143.

75. DeVisser MH, Messina JP, Moore NJ, Lusch DP, Maitima J. A dynamic species distribution model of *Glossina* subgenus *Morsitans*: The identification of tsetse reservoirs and refugia. 2010;1:art6.

76. Dhingra R, Jimenez V, Chang HH, Gambhir M, Fu JS, Liu Y, et al. Spatially-explicit simulation modeling of ecological response to climate change: Methodological considerations in predicting shifting population dynamics of infectious disease vectors. ISPRS international journal of geo-information. 2014;2:645-64.

77. Diuk-Wasser MA, Brown HE, Andreadis TG, Fish D. Modeling the spatial distribution of mosquito vectors for West Nile virus in Connecticut, USA. Vector borne and zoonotic diseases (Larchmont, NY). 2006;6:283-95.

78. Domsa C, Sandor AD, Mihalca AD. Climate change and species distribution: Possible scenarios for thermophilic ticks in Romania. Geospatial health. 2016;11:421.

79. Eastin MD, Delmelle E, Casas I, Wexler J, Self C. Intra- and interseasonal autoregressive prediction of dengue outbreaks using local weather and regional climate for a tropical environment in Colombia. The American journal of tropical medicine and hygiene. 2014;91:598-610.

80. Ebi KL, Hartman J, Chan N, McConnell J, Schlesinger M, Weyant J. Climate suitability for stable malaria transmission in Zimbabwe under different climate change scenarios. 2005;73:375-93.

81. Edlund S, Davis M, Douglas JV, Kershenbaum A, Waraporn N, Lessler J, et al. A global model of malaria climate sensitivity: Comparing malaria response to historic climate data based on simulation and officially reported malaria incidence. 2012;11.

82. Eisen L, Meyer AM, Eisen RJ. Climate-based model predicting acarological risk of encountering the human-biting adult life stage of *Dermacentor andersoni (Acari: Ixodidae)* in a key habitat type in Colorado. Journal of medical entomology. 2007;44:694-704.

83. Eisen RJ, Eisen L, Lane RS. Predicting density of *Ixodes pacificus* nymphs in dense woodlands in Mendocino County, California, based on geographic information systems and remote sensing versus field-derived data. The American journal of tropical medicine and hygiene. 2006;74:632-40.

84. Ekpo UF, Mafiana CF, Adeofun CO, Solarin AR, Idowu AB. Geographical information system and predictive risk maps of urinary schistosomiasis in Ogun State, Nigeria. BMC infectious diseases. 2008;8:74.

85. Elsheikh S, Patidar KC, Ouifki R. Analysis of a malaria model with a distributed delay. IMA J Appl Math. 2014;79:1139-60.

86. Erazo D, Cordovez J. Modeling the effects of palm-house proximity on the theoretical risk of Chagas disease transmission in a rural locality of the Orinoco basin, Colombia. Parasites and Vectors. 2016;9:1-5.

87. Erguler K, Smith-Unna SE, Waldock J, Proestos Y, Christophides GK, Lelieveld J, et al. Large-scale modelling of the environmentally-driven population dynamics of temperate *Aedes albopictus (Skuse)*. PLoS One. 2016;11:e0149282.

88. Erickson RA, Hayhoe K, Presley SM, Allen LJS, Long KR, Cox SB. Potential impacts of climate change on the ecology of dengue and its mosquito vector the Asian tiger mosquito (*Aedes albopictus*). 2012;7:034003.

89. Ermert V, Fink AH, Morse AP, Paeth H. The impact of regional climate change on malaria risk due to greenhouse forcing and land-use changes in tropical Africa. Environmental health perspectives. 2011;120:77-84.

90. Ermert V, Fink AH, Paeth H. The potential effects of climate change on malaria transmission in Africa using bias-corrected regionalised climate projections and a simple malaria seasonality model. Climatic Change. 2013;120:741-54.

91. Escobar LE, Qiao H, Peterson AT. Forecasting chikungunya spread in the Americas via data-driven empirical approaches. Parasites & vectors. 2016;9:112.

92. Esteva L, Yang HM. Assessing the effects of temperature and dengue virus load on dengue transmission. Journal of Biological Systems. 2015;23:527-54.

93. Estrada-Peña A. Diluting the dilution effect: A spatial Lyme model provides evidence for the importance of habitat fragmentation with regard to the risk of infection. Geospatial Health. 2009;3:143-55.

94. Estrada-Peña A, Martínez Avilés M, Muñoz Reoyo MJ. A population model to describe the distribution and seasonal dynamics of the tick *Hyalomma marginatum* in the Mediterranean Basin. Transboundary and Emerging Diseases. 2011;58:213-23.

95. Estrada-Peña A, Ruiz-Fons F, Acevedo P, Gortazar C, De la Fuente J. Factors driving the circulation and possible expansion of Crimean-Congo haemorrhagic fever virus in the western Palearctic. Journal of Applied Microbiology. 2013;114:278-86.

96. Estrada-Peña A, Sánchez N, Estrada-Sánchez A. An assessment of the distribution and spread of the tick *Hyalomma marginatum* in the western palearctic under different climate scenarios. Vector-Borne and Zoonotic Diseases. 2012;12:758-68.

97. Estrada-Pena A, Venzal JM. Climate niches of tick species in the Mediterranean region: Modeling of occurrence data, distributional constraints, and impact of climate change. Journal of medical entomology. 2007;44:1130-8.

98. Ewing DA, Cobbold CA, Purse BV, Nunn MA, White SM. Modelling the effect of temperature on the seasonal population dynamics of temperate mosquitoes. Journal of theoretical biology. 2016;400:65-79.

99. Ezanno P, Aubry-Kientz M, Arnoux S, Cailly P, L'Ambert G, Toty C, et al. A generic weather-driven model to predict mosquito population dynamics applied to species of Anopheles, Culex and Aedes genera of southern France. Preventive veterinary medicine. 2015;120:39-50.

100. Fabrizio MC, Schweigmann NJ, Bartoloni NJ. Modelling American trypanosomiasis in an endemic zone: Application to the initial spread of household infection in the Argentine Chaco. Zoonoses and public health. 2014;61:545-59.

101. Fatima SH, Atif S, Rasheed SB, Zaidi F, Hussain E. Species distribution modelling of *Aedes aegypti* in two dengue-endemic regions of Pakistan. Tropical medicine & international health : TM & IH. 2016;21:427-36.

102. Fernández MS, Salomón OD, Cavia R, Perez AA, Acardi SA, Guccione JD*. Lutzomyia longipalpis* spatial distribution and association with environmental variables in an urban focus of visceral leishmaniasis, Misiones, Argentina. Acta Tropica. 2010;114:81-7.

103. Ferreira CP, Pulino P, Yang HM, Takahashi LT. Controlling dispersal dynamics of *Aedes aegypti*. Math Popul Stud. 2006;13:215-36.

104. Ferro C, López M, Fuya P, Lugo L, Cordovez JM, González C. Spatial distribution of sand fly vectors and eco-epidemiology of cutaneous leishmaniasis transmission in Colombia. PLoS ONE. 2015;10.

105. Fischer D, Moeller P, Thomas SM, Naucke TJ, Beierkuhnlein C. Combining climatic projections and dispersal ability: A method for estimating the responses of sandfly vector species to climate change. Plos Neglected Tropical Diseases. 2011;5:13.

106. Fischer D, Thomas SM, Beierkuhnlein C. Temperature-derived potential for the establishment of phlebotomine sandflies and visceral leishmaniasis in Germany. Geospatial health. 2010;5:59-69.

107. Fischer D, Thomas SM, Beierkuhnlein C. Modelling climatic suitability and dispersal for disease vectors: the example of a phlebotomine sandfly in Europe. Spatial Statistics 2011: Mapping Global Change. 2011;7:164-9.

108. Fischer D, Thomas SM, Niemitz F, Reineking B, Beierkuhnlein C. Projection of climatic suitability for *Aedes albopictus Skuse (Culicidae)* in Europe under climate change conditions. Global & Planetary Change. 2011;78:54-64.

109. Fischer D, Thomas SM, Suk JE, Sudre B, Hess A, Tjaden NB, et al. Climate change effects on Chikungunya transmission in Europe: Geospatial analysis of vector's climatic suitability and virus' temperature requirements. 2013;12.

110. Foley DH, Klein TA, Kim HC, Wilkerson RC, Rueda LM. Malaria risk assessment for the Republic of Korea based on models of mosquito distribution. US Army Medical Department journal. 2008:46-53.

111. Foley P, Foley J. Modeling susceptible infective recovered dynamics and plague persistence in California rodent-flea communities. Vector borne and zoonotic diseases (Larchmont, NY). 2010;10:59-67.

112. Fuller DO, Ahumada ML, Quinones ML, Herrera S, Beier JC. Near-present and future distribution of *Anopheles albimanus* in Mesoamerica and the Caribbean Basin modeled with climate and topographic data. International journal of health geographics. 2012;11:13.

113. Fuller DO, Parenti MS, Hassan AN, Beier JC. Linking land cover and species distribution models to project potential ranges of malaria vectors: an example using *Anopheles arabiensis* in Sudan and Upper Egypt. Malaria journal. 2012;11:264.

114. Fuller DO, Troyo A, Beier JC. El Nĩo Southern Oscillation and vegetation dynamics as predictors of dengue fever cases in Costa Rica. Environmental Research Letters. 2009;4.

115. Gaff H, Schaefer E. Metapopulation models in tick-borne disease transmission modelling. Adv Exp Med Biol. 2010;673:51-65.

116. Gálvez R, Descalzo MA, Guerrero I, Miró G, Molina R. Mapping the current distribution and predicted spread of the leishmaniosis sand fly vector in the Madrid region (Spain) based on environmental variables and expected climate change. Vector-Borne and Zoonotic Diseases. 2011;11:799-806.

117. Gao D, Cosner C, Cantrell RS, Beier JC, Ruan S. Modeling the spatial spread of Rift Valley fever in Egypt. Bulletin of mathematical biology. 2013;75:523-42.

118. Gao M, Li X, Cao C, Zhang H, Li Q, Zhou H, et al. Spatial prediction and analysis of Himalayan marmot plague natural epidemic foci in China based on HJ-1 satellite data. 2010;53:8-15.

119. Garba SM, Gumel AB. Effect of cross-immunity on the transmission dynamics of two strains of dengue. Int J Comput Math. 2010;87:2361-84.

120. Gardner L, Sarkar S. A global airport-based risk model for the spread of dengue infection via the air transport network. PLoS One. 2013;8:e72129.

121. Gardner LM, Fajardo D, Waller ST, Wang O, Sarkar S. A predictive spatial model to quantify the risk of air-travel-associated dengue importation into the United States and Europe. Journal of tropical medicine. 2012;2012:103679.

122. Gardner LM, Sarkar S. Risk of dengue spread from the Philippines through international air travel. Transp Res Record. 2015:25-30.

123. Garza M, Feria Arroyo TP, Casillas EA, Sanchez-Cordero V, Rivaldi CL, Sarkar S. Projected future distributions of vectors of *Trypanosoma cruzi* in North America under climate change scenarios. PLoS neglected tropical diseases. 2014;8:e2818.

124. Gaudart J, Ghassani M, Mintsa J, Rachdi M, Waku J, Demongeot J. Demography and diffusion in epidemics: Malaria and Black Death spread. Acta Biotheoretica. 2010;58:277-305.

125. Gebre-Michael T, Malone JB, McNally K. Use of geographic information systems in the development of prediction models for onchocerciasis control in Ethiopia. Parassitologia. 2005;47:135-44.

126. Genchi C, Mortarino M, Rinaldi L, Cringoli G, Traldi G, Genchi M. Changing climate and changing vector-borne disease distribution: The example of Dirofilaria in Europe. Vet Parasitol. 2011;176:295-9.

127. Gething PW, Van Boeckel TP, Smith DL, Guerra CA, Patil AP, Snow RW, et al. Modelling the global constraints of temperature on transmission of *Plasmodium falciparum* and *P. vivax*. Parasites & vectors. 2011;4:92.

128. Gholamrezaei M, Mohebali M, Hanafi-Bojd AA, Sedaghat MM, Shirzadi MR. Ecological niche modeling of main reservoir hosts of zoonotic cutaneous leishmaniasis in Iran. Acta tropica. 2014;160:44-52.

129. Gomes E, Capinha C, Rocha J, Sousa C. Mapping risk of malaria transmission in mainland Portugal using a mathematical modelling approach. Plos One. 2016;11:17.

130. Gomez-Elipe A, Otero A, van Herp M, Aguirre-Jaime A. Forecasting malaria incidence based on monthly case reports and environmental factors in Karuzi, Burundi, 1997-2003. Malaria journal. 2007;6:129.

131. Gong H, DeGaetano AT, Harrington LC. Climate-based models for West Nile *Culex* mosquito vectors in the Northeastern US. International journal of biometeorology. 2010;55:435-46.

132. González C, Paz A, Ferro C. Predicted altitudinal shifts and reduced spatial distribution of *Leishmania infantum* vector species under climate change scenarios in Colombia. Acta Tropica. 2013;129:83-90.

133. Gonzalez C, Rebollar-Tellez EA, Ibanez-Bernal S, Becker-Fauser I, Martinez-Meyer E, Peterson AT, et al. Current knowledge of leishmania vectors in Mexico: How geographic distributions of species relate to transmission areas. Am J Trop Med Hyg. 2011;85:839-46.

134. González C, Wang O, Strutz SE, González-Salazar C, Sánchez-Cordero V, Sarkar S. Climate change and risk of leishmaniasis in North America: Predictions from ecological niche models of vector and reservoir species. PLoS Neglected Tropical Diseases. 2010;4.

135. Gouvêa MM. Time-spatial model on the dynamics of the proliferation of *Aedes aegypti*. Communications in Nonlinear Science and Numerical Simulation. 2017;44:130-43.

136. Griffin JT, Bhatt S, Sinka ME, Gething PW, Lynch M, Patouillard E, et al. Potential for reduction of burden and local elimination of malaria by reducing *Plasmodium falciparum* malaria transmission: A mathematical modelling study. The Lancet Infectious diseases. 2016;16:465-72.

137. Guimaraes RJ, Freitas CC, Dutra LV, Scholte RG, Martins-Bede FT, Fonseca FR, et al. A geoprocessing approach for studying and controlling schistosomiasis in the state of Minas Gerais, Brazil. Memorias do Instituto Oswaldo Cruz. 2010;105:524-31.

138. Guo JG, Vounatsou P, Cao CL, Utzinger J, Zhu HQ, Anderegg D, et al. A geographic information and remote sensing based model for prediction of *Oncomelania hupensis* habitats in the Poyang Lake area, China. Acta Trop. 2005;96:213-22.

139. Gurgel-Goncalves R, Cuba CA. Predicting the potential geographical distribution of *Rhodnius neglectus (Hemiptera, Reduviidae)* based on ecological niche modeling. Journal of medical entomology. 2009;46:952-60.

140. Hahn MB, Jarnevich CS, Monaghan AJ, Eisen RJ. Modeling the geographic distribution of *Ixodes scapularis* and *Ixodes pacificus (Acari: Ixodidae)* in the contiguous United States. Journal of medical entomology. 2016.

141. Halide H, Ridd P. A predictive model for dengue hemorrhagic fever epidemics. International journal of environmental health research. 2008;18:253-65.

142. Hall RJ, Brown LM, Altizer S. Modeling vector-borne disease risk in migratory animals under climate change. Integrative and comparative biology. 2016;56:353-64.

143. Hanafi-Bojd AA, Rassi Y, Yaghoobi-Ershadi MR, Haghdoost AA, Akhavan AA, Charrahy Z, et al. Predicted distribution of visceral leishmaniasis vectors (*Diptera: Psychodidae; Phlebotominae*) in Iran: A niche model study. Zoonoses and public health. 2015;62:644-54.

144. Hanafi-Bojd AA, Yaghoobi-Ershadi MR, Haghdoost AA, Akhavan AA, Rassi Y, Karimi A, et al. Modeling the distribution of cutaneous leishmaniasis vectors (*Psychodidae: Phlebotominae*) in Iran: A potential transmission in disease prone areas. J Med Entomol. 2015;52:557-65.

145. Hanandita W, Tampubolon G. Geography and social distribution of malaria in Indonesian Papua: A cross-sectional study. 2016;15.

146. Harrigan RJ, Thomassen HA, Buermann W, Smith TB. A continental risk assessment of West Nile virus under climate change. Global Change Biology. 2014;20:2417-25.

147. Hartemink NA, Davis SA, Reiter P, Hubálek Z, Heesterbeek JAP. Importance of bird-to-bird transmission for the establishment of West Nile virus. Vector-Borne and Zoonotic Diseases. 2007;7:575-84.

148. Hartfield M, Jane White KA, Kurtenbach K. The role of deer in facilitating the spatial spread of the pathogen *Borrelia burgdorferi*. Theoretical Ecology. 2011;4:27-36.

149. Heffernan JM, Lou Y, Wu J. Range expansion of *Ixodes scapularis* ticks and of *Borrelia burgdorferi* by migratory birds. Discrete and Continuous Dynamical Systems - Series B. 2014;19:3147-67.

150. Hii YL, Zhu H, Ng N, Ng LC, Rocklov J. Forecast of dengue incidence using temperature and rainfall. PLoS neglected tropical diseases. 2012;6:e1908.

151. Hill MP, Axford JK, Hoffmann AA. Predicting the spread of *Aedes albopictus* in Australia under current and future climates: Multiple approaches and datasets to incorporate potential evolutionary divergence. Austral Ecol. 2014;39:469-78.

152. Hincapie-Palacio D, Ospina J. Mathematical modeling of chikungunya fever control. Smart Biomedical and Physiological Sensor Technology Xii. 2015;9487.

153. Ho SH, Speldewinde P, Cook A. A Bayesian belief network for Murray Valley encephalitis virus risk assessment in Western Australia. International Journal of Health Geographics. 2016;15:19.

154. Ho SH, Speldewinde P, Cook A. Predicting arboviral disease emergence using Bayesian networks: A case study of dengue virus in Western Australia. Epidemiology and infection. 2016;145:54-66.

155. Hoch T, Breton E, Josse M, Deniz A, Guven E, Vatansever Z. Identifying main drivers and testing control strategies for CCHFV spread. Exp Appl Acarol. 2016;68:347-59.

156. Hoch T, Monnet Y, Agoulon A. Influence of host migration between woodland and pasture on the population dynamics of the tick *Ixodes ricinus*: A modelling approach. Ecological Modelling. 2010;221:1798-806.

157. Holt AC, Salkeld DJ, Fritz CL, Tucker JR, Gong P. Spatial analysis of plague in California: Niche modeling predictions of the current distribution and potential response to climate change. 2009;8.

158. Holy M, Schmidt G, Schroder W. Potential malaria outbreak in Germany due to climate warming: Risk modelling based on temperature measurements and regional climate models. Environmental science and pollution research international. 2010;18:428-35.

159. Hongoh V, Berrang-Ford L, Scott ME, Lindsay LR. Expanding geographical distribution of the mosquito, *Culex pipiens*, in Canada under climate change. Appl Geogr. 2012;33:53-62.

160. Houngbedji CA, Chammartin F, Yapi RB, Hurlimann E, N'Dri PB, Silue KD, et al. Spatial mapping and prediction of *Plasmodium falciparum* infection risk among school-aged children in Cote d'Ivoire. Parasites & vectors. 2016;9:494.

161. Hu W, Tong S, Mengersen K, Oldenburg B. Rainfall, mosquito density and the transmission of Ross River virus: A time-series forecasting model. 2006;196:505-14.

162. Ibañez-Justicia A, Cianci D. Modelling the spatial distribution of the nuisance mosquito species *Anopheles plumbeus (Diptera: Culicidae)* in the Netherlands. Parasites and Vectors. 2015;8.

163. Ibrahim A, Zin NAM, Ashaari NS. A simulation model using system dynamic for predicting dengue fever outbreak. 2011 Computation and Communication Technologies: 3rd International Conference on Advances in Computing, Control, and Telecommunication Technologies, ACT 2011 - Computer Science Series. 2011;1:128-30.

164. Illoldi-Rangel P, Rivaldi CL, Sissel B, Trout Fryxell R, Gordillo-Perez G, Rodriguez-Moreno A, et al. Species distribution models and ecological suitability analysis for potential tick vectors of Lyme disease in Mexico. Journal of tropical medicine. 2012;2012:959101.

165. Impoinvil DE, Ooi MH, Diggle PJ, Caminade C, Cardosa MJ, Morse AP, et al. The effect of vaccination coverage and climate on Japanese encephalitis in Sarawak, Malaysia. PLoS neglected tropical diseases. 2013;7:e2334.

166. Irvine MA, Reimer LJ, Njenga SM, Gunawardena S, Kelly-Hope L, Bockarie M, et al. Modelling strategies to break transmission of lymphatic filariasis - aggregation, adherence and vector competence greatly alter elimination. Parasites Vectors. 2015;8:19.

167. Isidoro C, Fachada N, Barata F, Rosa A. Agent-based model of dengue disease transmission by *Aedes aegypti* populations. Lecture Notes in Computer Science (including subseries Lecture Notes in Artificial Intelligence and Lecture Notes in Bioinformatics). 2011;5777 LNAI:345-52.

168. Jacintho LFO, Batista AFM, Ruas TL, Marietto MGB, Silva FA. An agent-based model for the spread of the dengue fever: A Swarm platform simulation approach. Spring Simulation Multiconference 2010, SpringSim'10. 2010.

169. Jafaruddin J, Puspita J, Nuraini N, Soewono E. A dynamical model for transmission of West Nile virus in chicken-mosquito interaction. Journal of Mathematical and Fundamental Sciences. 2014;46:290-300.

170. Jamil JM, Shaharanee INM. An innovative forecasting and dashboard system for Malaysian dengue trends. Proceedings of the International Conference on Applied Science and Technology 2016. 2016;1761.

171. Jaya I, Abdullah AS, Hermawan E, Ruchjana BN. Bayesian spatial modeling and mapping of dengue fever: A case study of dengue fever in the city of Bandung, Indonesia. Int J Appl Math Stat. 2016;54:94-103.

172. Jensen S, Van Peursem D. Controlling plague among prairie dogs: A two colony epidemiological model. WSEAS Transactions on Biology and Biomedicine. 2013;10:67-78.

173. Johansson MA, Arana-Vizcarrondo N, Biggerstaff BJ, Gallagher N, Marano N, Staples JE. Assessing the risk of international spread of yellow fever virus: A mathematical analysis of an urban outbreak in Asuncion, 2008. Am J Trop Med Hyg. 2012;86:349-58.

174. Johansson MA, Powers AM, Pesik N, Cohen NJ, Staples JE. Nowcasting the spread of chikungunya virus in the Americas. PLoS One. 2014;9:e104915.

175. Johnson TL, Bjork JK, Neitzel DF, Dorr FM, Schiffman EK, Eisen RJ. Habitat suitability model for the distribution of *Ixodes scapularis (Acari: Ixodidae)* in Minnesota. Journal of medical entomology. 2016;53:598-606.

176. Jones EO, Webb SD, Ruiz-Fons FJ, Albon S, Gilbert L. The effect of landscape heterogeneity and host movement on a tick-borne pathogen. Theoretical Ecology. 2011;4:435-48.

177. Juan P, Díaz-Avalos C, Mejía-Domínguez NR, Mateu J. Hierarchical spatial modeling of the presence of Chagas disease insect vectors in Argentina. A comparative approach. Stochastic Environmental Research and Risk Assessment. 2016:1-19.

178. Kaga T, Ohta S. Ecophysiological and climatological effects on distribution of vector species and malaria incidence in India. International Journal of Environmental Research and Public Health. 2012;9:4704-14.

179. Karl S, Halder N, Kelso JK, Ritchie SA, Milne GJ. A spatial simulation model for dengue virus infection in urban areas. BMC Infect Dis. 2014;14:17.

180. Karl S, White MT, Milne GJ, Gurarie D, Hay SI, Barry AE, et al. Spatial effects on the multiplicity of *Plasmodium falciparum* infections. Plos One. 2016;11:20.

181. Kartashev V, Afonin A, González-Miguel J, Sepúlveda R, Simón L, Morchón R, et al. Regional warming and emerging vector-borne zoonotic dirofilariosis in the Russian Federation, Ukraine, and other post-soviet states from 1981 to 2011 and projection by 2030. BioMed Research International. 2014;2014.

182. Kashiwada M, Ohta S. Modeling the spatio-temporal distribution of the *Anopheles* mosquito based on life history and surface water conditions. 2010;3:29-40.

183. Kassem HA, Siri J, Kamal HA, Wilson ML. Environmental factors underlying spatial patterns of sand flies (*Diptera: Psychodidae*) associated with leishmaniasis in southern Sinai, Egypt. Acta Tropica. 2012;123:8-15.

184. Khatchikian C, Sangermano F, Kendell D, Livdahl T. Evaluation of species distribution model algorithms for fine-scale container-breeding mosquito risk prediction. Medical and veterinary entomology. 2011;25:268-75.

185. Khatchikian CE, Prusinski M, Stone M, Backenson PB, Wang IN, Levy MZ, et al. Geographical and environmental factors driving the increase in the Lyme disease vector *Ixodes scapularis.* Ecosphere (Washington, DC). 2012;3.

186. Khormi HM, Kumar L. Assessing the risk for dengue fever based on socioeconomic and environmental variables in a geographical information system environment. Geospatial Health. 2012;6:171-6.

187. Khormi HM, Kumar L. Climate change and the potential global distribution of *Aedes aegypti*: Spatial modelling using GIS and CLIMEX. Geospatial health. 2014;8:405-15.

188. Khormi HM, Kumar L. Future malaria spatial pattern based on the potential global warming impact in South and Southeast Asia. Geospatial Health. 2016;11:290-8.

189. Khormi HM, Lalit K, Elzahrany RA. Modeling spatio-temporal risk changes in the incidence of dengue fever in Saudi Arabia: A geographical information system case study. 2011;6:77-84.

190. Kilpatrick AM, Daszak P, Goodman SJ, Rogg H, Kramer LD, Cedeño V, et al. Predicting pathogen introduction: West Nile virus spread to Galápagos. Conservation Biology. 2006;20:1224-31.

191. Kim Y-M, Park J-W, Cheong H-K. Estimated effect of climatic variables on the transmission of *Plasmodium vivax* malaria in the Republic of Korea. Environmental Health Perspectives. 2012;120:1314-9.

192. Knerer G, Currie CSM, Brailsford SC. Impact of combined vector-control and vaccination strategies on transmission dynamics of dengue fever: A model-based analysis. Health Care Management Science. 2013:1-13.

193. Konrad SK, Miller SN. A temperature-limited assessment of the risk of Rift Valley fever transmission and establishment in the continental United States of America. Geospatial Health. 2012;6:161-70.

194. Konrad SK, Miller SN, Reeves WK. A spatially explicit degree-day model of Rift Valley fever transmission risk in the continental United States. GeoJournal. 2011;76:257-66.

195. Korenromp E, Mahiané G, Hamilton M, Pretorius C, Cibulskis R, Lauer J, et al. Malaria intervention scale-up in Africa: Effectiveness predictions for health programme planning tools, based on dynamic transmission modelling. Malaria Journal. 2016;15.

196. Kulkarni MA, Desrochers RE, Kerr JT. High resolution niche models of malaria vectors in northern Tanzania: a new capacity to predict malaria risk? PLoS One. 2010;5:e9396.

197. Kursah MB. Modelling malaria susceptibility using geographic information system. GeoJournal. 2016:1-11.

198. Laperriere V, Brugger K, Rubel F. Cross-scale modeling of a vector-borne disease, from the individual to the metapopulation: The seasonal dynamics of sylvatic plague in Kazakhstan. 2016;342:34-48.

199. Laporta GZ, Linton YM, Wilkerson RC, Bergo ES, Nagaki SS, Sant'Ana DC, et al. Malaria vectors in South America: Current and future scenarios. Parasites & vectors. 2015;8:426.

200. Laporta GZ, Ramos DG, Ribeiro MC, Sallum MA. Habitat suitability of *Anopheles* vector species and association with human malaria in the Atlantic Forest in south-eastern Brazil. Memorias do Instituto Oswaldo Cruz. 2011;106 Suppl 1:239-45.

201. Leedale J, Jones AE, Caminade C, Morse AP. A dynamic, climate-driven model of Rift Valley fever. Geospatial Health. 2016;11:78-93.

202. Leedale J, Tompkins AM, Caminade C, Jones AE, Nikulin G, Morse AP. Projecting malaria hazard from climate change in eastern Africa using large ensembles to estimate uncertainty. Geospatial Health. 2016;11:102-14.

203. Leedale J, Tompkins AM, Caminade C, Jones AE, Nikulin G, Morse AP. Projecting malaria hazard from climate change in eastern Africa using large ensembles to estimate uncertainty. Special Issue: Health, environmental change and adaptive capacity: Mapping, examining and anticipating future risks of water-related vector-borne diseases in Eastern Africa. 2016;11:102-14.

204. Leighton PA, Koffi JK, Pelcat Y, Lindsay LR, Ogden NH. Predicting the speed of tick invasion: An empirical model of range expansion for the Lyme disease vector *Ixodes scapularis* in Canada. Journal of Applied Ecology. 2012;49:457-64.

205. Lewnard JA, Jirmanus L, Júnior NN, Machado PR, Glesby MJ, Ko AI, et al. Forecasting temporal dynamics of cutaneous leishmaniasis in Northeast Brazil. PLoS Neglected Tropical Diseases. 2014;8.

206. Li S, Gilbert L, Harrison PA, Rounsevell MD. Modelling the seasonality of Lyme disease risk and the potential impacts of a warming climate within the heterogeneous landscapes of Scotland. Journal of the Royal Society, Interface. 2016;13.

207. Liang S, Spear RC, Seto E, Hubbard A, Qiu D. A multi-group model of *Schistosoma japonicum* transmission dynamics and control: model calibration and control prediction. 2005;10:263-78.

208. Lin H, Liu T, Song T, Lin L, Xiao J, Lin J, et al. Community involvement in dengue outbreak control: An integrated rigorous intervention strategy. PLoS neglected tropical diseases. 2016;10:e0004919.

209. Lindsay SW, Hole DG, Hutchinson RA, Richards SA, Willis SG. Assessing the future threat from *vivax* malaria in the United Kingdom using two markedly different modelling approaches. Malaria journal. 2010;9:70.

210. Little E, Campbell SR, Shaman J. Development and validation of a climate-based ensemble prediction model for West Nile Virus infection rates in *Culex* mosquitoes, Suffolk County, New York. Parasites & vectors. 2016;9:443.

211. Liu R, Shuai J, Wu J, Zhu H. Modeling spatial spread of West Nile virus and impact of directional dispersal of birds. Mathematical Biosciences and Engineering. 2006;3:145-60.

212. Liu-Helmersson J, Quam M, Wilder-Smith A, Stenlund H, Ebi K, Massad E, et al. Climate change and *Aedes* vectors: 21st century projections for dengue transmission in Europe. EBioMedicine. 2016;7:267-77.

213. Liu-Helmersson J, Stenlund H, Wilder-Smith A, Rocklöv J. Vectorial capacity of *Aedes aegypti*: Effects of temperature and implications for global dengue epidemic potential. PLoS ONE. 2014;9.

214. Lowe R, Barcellos C, Coelho CAS, Bailey TC, Coelho GE, Graham R, et al. Dengue outlook for the World Cup in Brazil: An early warning model framework driven by real-time seasonal climate forecasts. The Lancet Infectious Diseases. 2014;14:619-26.

215. Luz PM, Lima-Camara TN, Bruno RV, e Castro MG, Sorgine MHF, Lourenço-de-Oliveira R, et al. Potential impact of a presumed increase in the biting activity of dengue-virus-infected *Aedes aegypti (Diptera: Culicidae)* females on virus transmission dynamics. Memorias do Instituto Oswaldo Cruz. 2011;106:755-8.

216. Lv S, Zhang Y, Steinmann P, Yang GJ, Yang K, Zhou XN, et al. The emergence of angiostrongyliasis in the People's Republic of China: The interplay between invasive snails, climate change and transmission dynamics. Freshw Biol. 2011;56:717-34.

217. Machault V, Vignolles C, Pages F, Gadiaga L, Tourre YM, Gaye A, et al. Risk mapping of *Anopheles gambiae s.l.* densities using remotely-sensed environmental and meteorological data in an urban area: Dakar, Senegal. PLoS One. 2012;7:e50674.

218. MacMillan K, Monaghan AJ, Apangu T, Griffith KS, Mead PS, Acayo S, et al. Climate predictors of the spatial distribution of human plague cases in the West Nile region of Uganda. Am J Trop Med Hyg. 2012;86:514-23.

219. Madhav NK, Brownstein JS, Tsao JI, Fish D. A dispersal model for the range expansion of blacklegged tick (*Acari: Ixodidae*). Journal of medical entomology. 2004;41:842-52.

220. Maidana NA, Yang HM. Assessing the spatial propagation of West Nile virus. Biomat 2007. 2008:259-73.

221. Maidana NA, Yang HM. Spatial spreading of West Nile virus described by traveling waves. Journal of Theoretical Biology. 2009;258:403-17.

222. Maidana NA, Yang HM. Dynamic of West Nile virus transmission considering several coexisting avian populations. Math Comput Model. 2011;53:1247-60.

223. Mak S, Morshed M, Henry B. Ecological niche modeling of Lyme disease in British Columbia, Canada. 2010;47:99-105.

224. Maneerat S, Daudé E. A spatial agent-based simulation model of the dengue vector *Aedes aegypti* to explore its population dynamics in urban areas. Ecological Modelling. 2016;333:66-78.

225. Mangal TD, Paterson S, Fenton A. Predicting the impact of long-term temperature changes on the epidemiology and control of schistosomiasis: A mechanistic model. PLoS One. 2008.

226. Manore CA, Davis J, Christofferson RC, Wesson D, Hyman JM, Mores CN. Towards an early warning system for forecasting human West Nile virus incidence. PLoS currents. 2014;6.

227. Manore CA, Hickmann KS, Hyman JM, Foppa IM, Davis JK, Wesson DM, et al. A network-patch methodology for adapting agent-based models for directly transmitted disease to mosquito-borne disease. J Biol Dyn. 2015;9:52-72.

228. Manore CA, Hickmann KS, Xu S, Wearing HJ, Hyman JM. Comparing dengue and chikungunya emergence and endemic transmission in *A. aegypti* and *A. albopictus*. Journal of Theoretical Biology. 2014;356:174-91.

229. Manyangadze T, Chimbari MJ, Gebreslasie M, Ceccato P, Mukaratirwa S. Modelling the spatial and seasonal distribution of suitable habitats of schistosomiasis intermediate host snails using Maxent in Ndumo area, KwaZulu-Natal Province, South Africa. Parasites & vectors. 2016;9:572.

230. Marcantonio M, Metz M, Baldacchino F, Arnoldi D, Montarsi F, Capelli G, et al. First assessment of potential distribution and dispersal capacity of the emerging invasive mosquito *Aedes koreicus* in Northeast Italy. Parasites Vectors. 2016;9:19.

231. Martcheva M, Hoppensteadt F. India’s approach to eliminating *Plasmodium falciparum* malaria: A modeling perspective. Journal of Biological Systems. 2010;18:867-91.

232. Martins-Bede FT, Dutra LV, Freitas CC, Guimaraes RJ, Amaral RS, Drummond SC, et al. Schistosomiasis risk mapping in the state of Minas Gerais, Brazil, using a decision tree approach, remote sensing data and sociological indicators. Memorias do Instituto Oswaldo Cruz. 2010;105:541-8.

233. Martins-Bede FT, Freitas CC, Dutra LV, Sandri SA, Fonseca FR, Drummond IN, et al. Risk mapping of schistosomiasis in Minas Gerais, Brazil, using MODIS and socioeconomic spatial data. IEEE Transactions on Geoscience and Remote Sensing. 2009;47:3899-908.

234. Massad E, Tan SH, Khan K, Wilder-Smith A. Estimated Zika virus importations to Europe by travellers from Brazil. Global Health Action. 2016;9:1-7.

235. Masuelli S, Rotela CH, Lamfri M, Scavuzzo CM. *Aedes aegypti* spatio-temporal modelling based on non homogeneous environment derived from remote sensors information. Proceedings, 32nd International Symposium on Remote Sensing of Environment: Sustainable Development Through Global Earth Observations. 2007.

236. Matawa F, Murwira A, Zengeya FM, Atkinson PM. Modelling the spatial-temporal distribution of tsetse (*Glossina pallidipes*) as a function of topography and vegetation greenness in the Zambezi Valley of Zimbabwe. Appl Geogr. 2016;76:198-206.

237. McCreesh N, Nikulin G, Booth M. Predicting the effects of climate change on *Schistosoma mansoni* transmission in eastern Africa. Parasites & vectors. 2015;8:4.

238. McMahon BH, Manore CA, Hyman JM, LaBute MX, Fair JM. Coupling vector-host dynamics with weather geography and mitigation measures to model Rift Valley fever in Africa. Math Model Nat Phenom. 2014;9:161-77.

239. Mecoli M, De Angelis V, Brailsford SC. Modelling the risk of mosquito-borne diseases by system dynamics: The case of human travel between different geographic regions. Conference Proceedings - IEEE International Conference on Systems, Man and Cybernetics. 2010:19-25.

240. Medley KA. Niche shifts during the global invasion of the Asian tiger mosquito, *Aedes albopictus Skuse (Culicidae)*, revealed by reciprocal distribution models. Glob Ecol Biogeogr. 2010;19:122-33.

241. Melaun C, Werblow A, Cunze S, Zotzmann S, Koch LK, Mehlhorn H, et al. Modeling of the putative distribution of the arbovirus vector *Ochlerotatus japonicus japonicus (Diptera: Culicidae)* in Germany. Parasitology Research. 2015;114:1051-61.

242. Meneguzzi VC, Santos CB, Leite GR, Fux B, Falqueto A. Environmental niche modelling of *Phlebotomine* sand flies and cutaneous leishmaniasis *identifies Lutzomyia intermedia* as the main vector species in Southeastern Brazil. PLoS One. 2016;11:e0164580.

243. Mesk M, Mandjoub T, Gourbiere S, Rabinovich JE, Menu F. Invasion speeds of *Triatoma dimidiata*, vector of Chagas disease: An application of orthogonal polynomials method. Journal of Theoretical Biology. 2016;395:126-43.

244. Messina JP, Kraemer MUG, Brady OJ, Pigott DM, Shearer FM, Weiss DJ, et al. Mapping global environmental suitability for Zika virus. eLife. 2016;5:19.

245. Messina JP, Moore NJ, DeVisser MH, McCord PF, Walker ED. Climate change and risk projection: Dynamic spatial models of tsetse and African trypanosomiasis in Kenya. Ann Assoc Am Geogr. 2012;102:1038-48.

246. Miller RH, Masuoka P, Klein TA, Kim HC, Somer T, Grieco J. Ecological niche modeling to estimate the distribution of Japanese encephalitis virus in Asia. PLoS neglected tropical diseases. 2012;6:e1678.

247. Miron RE, Giordano GA, Kealey AD, Smith RJ. Multiseason transmission for Rift Valley fever in North America. Math Popul Stud. 2016;23:71-94.

248. Moffett A, Shackelford N, Sarkar S. Malaria in Africa: Vector species' niche models and relative risk maps. PLoS One. 2007;2:e824.

249. Mohammed Baba A, Yahya Abu H, Farah Aini A. Modeling and optimal control of *Plasmodium knowlesi* malaria spread from infected humans to mosquitoes. 2014;4:4482-501.

250. Mokhtari M, Miri M, Nikoonahad A, Jalilian A, Naserifar R, Ghaffari HR, et al. Cutaneous leishmaniasis prevalence and morbidity based on environmental factors in Ilam, Iran: Spatial analysis and land use regression models. Acta Tropica. 2016;163:90-7.

251. Moo-Llanes D, Ibarra-Cerdena CN, Rebollar-Tellez EA, Ibanez-Bernal S, Gonzalez C, Ramsey JM. Current and future niche of North and Central American sand flies (*Diptera: psychodidae*) in climate change scenarios. PLoS neglected tropical diseases. 2013;7:e2421.

252. Moore S, Shrestha S, Tomlinson KW, Vuong H. Predicting the effect of climate change on African trypanosomiasis: Integrating epidemiology with parasite and vector biology. J R Soc Interface. 2012;9:817-30.

253. Moraga P, Cano J, Baggaley RF, Gyapong JO, Njenga SM, Nikolay B, et al. Modelling the distribution and transmission intensity of lymphatic filariasis in sub-Saharan Africa prior to scaling up interventions: integrated use of geostatistical and mathematical modelling. Parasites Vectors. 2015;8:16.

254. Mordecai EA, Paaijmans KP, Johnson LR, Balzer C, Ben-Horin T, e Moor E, et al. Optimal temperature for malaria transmission is dramatically lower than previously predicted. Ecology letters. 2012;16:22-30.

255. Morin CW, Comrie AC. Modeled response of the West Nile virus vector *Culex quinquefasciatus* to changing climate using the dynamic mosquito simulation model. International journal of biometeorology. 2010;54:517-29.

256. Morin CW, Comrie AC. Regional and seasonal response of a West Nile virus vector to climate change. Proceedings of the National Academy of Sciences of the United States of America. 2013;110:15620-5.

257. Moulay D, Pigné Y. A metapopulation model for chikungunya including populations mobility on a large-scale network. Journal of Theoretical Biology. 2013;318:129-39.

258. Moyes CL, Shearer FM, Huang Z, Wiebe A, Gibson HS, Nijman V, et al. Predicting the geographical distributions of the macaque hosts and mosquito vectors of *Plasmodium knowlesi* malaria in forested and non-forested areas. Parasites & vectors. 2016;9:242.

259. Mpeshe SC, Luboobi LS, Nkansah-Gyekye Y. Modeling the impact of climate change on the dynamics of Rift Valley fever. Computational and mathematical methods in medicine. 2014;2014:627586.

260. Musa MI, Shohaimi S, Hashim NR, Krishnarajah I. A climate distribution model of malaria transmission in Sudan. Geospatial Health. 2013;7:27-36.

261. Mweya CN, Kimera SI, Kija JB, Mboera LE. Predicting distribution of *Aedes aegypti* and *Culex pipiens* complex, potential vectors of Rift Valley fever virus in relation to disease epidemics in East Africa. Infection ecology & epidemiology. 2013;3.

262. Naeem M, Alahmed AM, Kheir SM, Sallam MF. Spatial distribution modeling of *Stegomyia aegypti* and *Culex tritaeniorhynchus (Diptera: Culicidae)* in Al-bahah Province, Kingdom of Saudi Arabia. Trop Biomed. 2016;33:295-310.

263. Nah K, Mizumoto K, Miyamatsu Y, Yasuda Y, Kinoshita R, Nishiura H. Estimating risks of importation and local transmission of Zika virus infection. PeerJ. 2016;4:15.

264. Nakazawa Y, Williams R, Peterson AT, Mead P, Staples E, Gage KL. Climate change effects on plague and tularemia in the United States. Vector borne and zoonotic diseases (Larchmont, NY). 2007;7:529-40.

265. Ndeffo-Mbah ML, Durham DP, Skrip LA, Nsoesie EO, Brownstein JS, Fish D, et al. Evaluating the effectiveness of localized control strategies to curtail chikungunya. Sci Rep. 2016;6:9.

266. Ndii MZ, Allingham D, Hickson RI, Glass K. The effect of Wolbachia on dengue outbreaks when dengue is repeatedly introduced. Theor Popul Biol. 2016;111:9-15.

267. Ndii MZ, Hickson RI, Allingham D, Mercer GN. Modelling the transmission dynamics of dengue in the presence of Wolbachia. Math Biosci. 2015;262:157-66.

268. Neteler M, Metz M, Rocchini D, Rizzoli A, Flacio E, Engeler L, et al. Is Switzerland suitable for the invasion of *Aedes albopictus*? PLoS ONE. 2013;8.

269. Neteler M, Roiz D, Rocchini D, Castellani C, Rizzoli A. Terra and aqua satellites track tiger mosquito invasion: Modelling the potential distribution of *Aedes albopictus* in north-eastern Italy. International Journal of Health Geographics. 2011;10:13.

270. Newth D, Gunasekera D. Climate change and the effects of dengue upon Australia: An analysis of health impacts and costs. IOP Conf Ser Earth Envir Sci. 2010;11.

271. Ng V, Dear K, Harley D, McMichael A. Analysis and prediction of Ross River virus transmission in New South Wales, Australia. Vector-Borne and Zoonotic Diseases. 2014;14:422-38.

272. Ngarakana-Gwasira ET, Bhunu CP, Masocha M, Mashonjowa E. Assessing the role of climate change in malaria transmission in Africa. Malaria research and treatment. 2016;2016:7104291.

273. Nieto P, Malone JB, Bavia ME. Ecological niche modeling for visceral leishmaniasis in the state of Bahia, Brazil, using genetic algorithm for rule-set prediction and growing degree day-water budget analysis. Geospatial health. 2008;1:115-26.

274. Nijhof S, Cox J, e Vries P, Bradley DJ, McMichael AJ, Martens P, et al. Climate change and future populations at risk of malaria. Global Environmental Change Part A: Human & Policy Dimensions. 1999;9:S89.

275. Nikolov M, Bever CA, Upfill-Brown A, Hamainza B, Miller JM, Eckhoff PA, et al. Malaria elimination campaigns in the Lake Kariba region of Zambia: A spatial dynamical model. PLoS computational biology. 2016;12:e1005192.

276. Niu T, Gaff HD, Papelis YE, Hartley DM. An epidemiological model of Rift Valley fever with spatial dynamics. Computational and mathematical methods in medicine. 2012;2012:138757.

277. Nsoesie EO, Kraemer MUG, Golding N, Pigott DM, Brady OJ, Moyes CL, et al. Global distribution and environmental suitability for chikungunya virus, 1952 to 2015. Eurosurveillance. 2016;21.

278. Obsomer V, Defourny P, Coosemans M. Predicted distribution of major malaria vectors belonging to the *Anopheles dirus* complex in Asia: Ecological niche and environmental influences. PLoS One. 2012;7:e50475.

279. Ochieng AO, Nanyingi M, Kipruto E, Ondiba IM, Amimo FA, Oludhe C, et al. Ecological niche modelling of Rift Valley fever virus vectors in Baringo, Kenya. Infection ecology & epidemiology. 2016;6:32322.

280. Ogden NH, Bigras-Poulin M, Hanincová K, Maarouf A, Callaghan CJ, Kurtenbach K. Projected effects of climate change on tick phenology and fitness of pathogens transmitted by the North American tick *Ixodes scapularis*. Journal of Theoretical Biology. 2008;254:621-32.

281. Ogden NH, Bigras-Poulin M, O'Callaghan CJ, Barker IK, Lindsay LR, Maarouf A, et al. A dynamic population model to investigate effects of climate on geographic range and seasonality of the tick *Ixodes scapularis*. International journal for parasitology. 2005;35:375-89.

282. Ogden NH, Lindsay LR, Leighton PA, Sheppard A. Predicting the rate of invasion of the agent of Lyme disease *Borrelia burgdorferi*. Journal of Applied Ecology. 2013;50:510-8.

283. Ogden NH, Maarouf A, Barker IK, Bigras-Poulin M, Lindsay LR, Morshed MG, et al. Climate change and the potential for range expansion of the Lyme disease vector *Ixodes scapularis* in Canada. International journal for parasitology. 2005;36:63-70.

284. Ogden NH, Milka R, Caminade C, Gachon P. Recent and projected future climatic suitability of North America for the Asian tiger mosquito *Aedes albopictus*. Parasites & vectors. 2014;7:532.

285. Ogden NH, Radojević M, Wu X, Duvvuri VR, Leighton PA, Wu J. Estimated effects of projected climate change on the basic reproductive number of the Lyme disease vector *Ixodes scapularis*. Environmental Health Perspectives. 2014;122:631-8.

286. Ogden NH, St.-Onge L, Barker IK, Brazeau S, Bigras-Poulin M, Charron DF, et al. Risk maps for range expansion of the Lyme disease vector, *Ixodes scapularis*, in Canada now and with climate change. International Journal of Health Geographics. 2008;7.

287. Ohemeng FD, Mukherjee F. Modelling the spatial distribution of the *Anopheles* mosquito for malaria risk zoning using remote sensing and GIS: A case study in the Zambezi Basin, Zimbabwe. International Journal of Applied Geospatial Research. 2015;6:7-20.

288. Ohta S, Kaga T. Effect of climate on malarial vector distribution in monsoon Asia: Coupled model for ecophysiological and climatological distribution of mosquito generations (ECD-mg). Clim Res. 2012.

289. Ohta S, Kaga T. Effect of irrigation systems on temporal distribution of malaria vectors in semi-arid regions. 2014;58:349-59.

290. Oki M, Sunahara T, Hashizume M, Yamamoto T. Optimal timing of insecticide fogging to minimize dengue cases: Modeling dengue transmission among various seasonalities and transmission intensities. Plos Neglected Tropical Diseases. 2011;5:7.

291. Olgen MK, Ozbel Y, Balcioglu IC, Demir S, Simsek F, Toz SO, et al. A new approach for determining the spatial risk levels for visceral and cutaneous leishmaniasis related with the distribution of vector species in western part of Turkey using geographical information systems and remote sensing. Kafkas Univ Vet Fak Derg. 2012;18:A77-A84.

292. Olwoch JM, Reyers B, Jaarsveld ASv. Host-parasite distribution patterns under simulated climate: Implications for tick-borne diseases. Special Issue: African climate and applications. 2009;29:993-1000.

293. Olwoch JM, Van Jaarsveld AS, Scholtz CH, Horak IG. Climate change and the genus *Rhipicephalus (Acari: Ixodidae)* in Africa. The Onderstepoort journal of veterinary research. 2007;74:45-72.

294. Ortiz PL, Rivero A, Linares Y, Pérez A, Vázquez JR. Spatial models for prediction and early warning of *Aedes aegypti* proliferation from data on climate change and variability in Cuba. MEDICC Review. 2015;17:20-8.

295. Otero M, Barmak DH, Dorso CO, Solari HG, Natiello MA. Modeling dengue outbreaks. Math Biosci. 2011;232:87-95.

296. Paaijmans K, Blanford J, Crane R, Mann M, Ning L, Schreiber K, et al. Downscaling reveals diverse effects of anthropogenic climate warming on the potential for local environments to support malaria transmission. Climatic Change. 2014;125:479-88.

297. Padmanabha H, Correa F, Rubio C, Baeza A, Osorio S, Mendez J, et al. Human social behavior and demography drive patterns of fine-scale dengue transmission in endemic areas of Colombia. Plos One. 2015;10:21.

298. Pandey A, Medlock J. The introduction of dengue vaccine may temporarily cause large spikes in prevalence. Epidemiol Infect. 2015;143:1276-86.

299. Parham PE, Michael E. Modeling the effects of weather and climate change on malaria transmission. Environmental Health Perspectives. 2010;118:620-6.

300. Parham PE, Michael E. Modelling climate change and malaria transmission. Adv Exp Med Biol. 2010;673:184-99.

301. Parra-Henao G, Quiros-Gomez O, Jaramillo ON, Cardona AS. Environmental determinants of the distribution of chagas disease vector *Triatoma dimidiata* in Colombia. The American journal of tropical medicine and hygiene. 2016;94:767-74.

302. Pedersen UB, Stendel M, Midzi N, Mduluza T, Soko W, Stensgaard AS, et al. Modelling climate change impact on the spatial distribution of fresh water snails hosting trematodes in Zimbabwe. Parasites & vectors. 2014;7:536.

303. Perkins TA, Siraj AS, Ruktanonchai CW, Kraemer MUG, Tatem AJ. Model-based projections of Zika virus infections in childbearing women in the Americas. Nat Microbiol. 2016;1:7.

304. Peterson AT. Shifting suitability for malaria vectors across Africa with warming climates. BMC infectious diseases. 2009;9:59.

305. Peterson AT, Campbell LP. Global potential distribution of the mosquito *Aedes notoscriptus*, a new alien species in the United States. 2015;40:191-4.

306. Peterson AT, Shaw J. *Lutzomyia* vectors for cutaneous leishmaniasis in Southern Brazil: Ecological niche models, predicted geographic distributions, and climate change effects. International journal for parasitology. 2003;33:919-31.

307. Peterson AT, Vieglais DA, Andreasen JK. Migratory birds modeled as critical transport agents for West Nile virus in North America. Vector borne and zoonotic diseases (Larchmont, NY). 2003;3:27-37.

308. Peterson JK, Bartsch SM, Lee BY, Dobson AP. Broad patterns in domestic vector-borne *Trypanosoma cruzi* transmission dynamics: Synanthropic animals and vector control quantitative analysis of strategies to achieve the 2020 goals for neglected tropical diseases: Where are we now? Parasites and Vectors. 2015;8.

309. Pikula J, Beklova M, Holesovska Z, Treml F. Prediction of possible distribution of tularemia in the Czech Republic. 2004;49:61-4.

310. Pinto C, Rocha D. A modified mathematical model for malaria transmission under control strategies. IEEE 4th International Conference on Nonlinear Science and Complexity, NSC 2012 - Proceedings. 2012:25-32.

311. Pizzitutti F, Pan W, Barbieri A, Miranda JJ, Feingold B, Guedes GR, et al. A validated agent-based model to study the spatial and temporal heterogeneities of malaria incidence in the rainforest environment. Malaria Journal. 2015;14:19.

312. Polwiang S. The estimation of imported dengue virus from thailand. J Travel Med. 2015;22:194-9.

313. Ponçon N, Tran A, Toty C, Luty AJF, Fontenille D. A quantitative risk assessment approach for mosquito-borne diseases: Malaria re-emergence in southern France. Malaria Journal. 2008;7.

314. Popov IO, Yasyukevich VV. The taiga tick *Ixodes persulcatus*: Propagation under climate change conditions in the 21st century. 2014;39:558-63.

315. Porretta D, Mastrantonio V, Amendolia S, Gaiarsa S, Epis S, Genchi C, et al. Effects of global changes on the climatic niche of the tick *Ixodes ricinus* inferred by species distribution modelling. Parasites & vectors. 2013;6:271.

316. Pradier S, Leblond A, Durand B. Land cover, landscape structure, and West Nile virus circulation in southern France. Vector borne and zoonotic diseases (Larchmont, NY). 2008;8:253-63.

317. Proestos Y, Christophides GK, Erguler K, Tanarhte M, Waldock J, Lelieveld J. Present and future projections of habitat suitability of the Asian tiger mosquito, a vector of viral pathogens, from global climate simulation. Philos Trans R Soc B-Biol Sci. 2015;370:16.

318. Quintana M, Salomón O, Guerra R, Lizarralde De Grosso M, Fuenzalida A. Phlebotominae of epidemiological importance in cutaneous leishmaniasis in northwestern Argentina: Risk maps and ecological niche models. Medical and Veterinary Entomology. 2013;27:39-48.

319. Qviller L, Viljugrein H, Loe LE, Meisingset EL, Mysterud A. The influence of red deer space use on the distribution of *Ixodes ricinus* ticks in the landscape. Parasites & vectors. 2016;9:545.

320. Rajabi M, Pilesjo P, Shirzadi MR, Fadaei R, Mansourian A. A spatially explicit agent-based modeling approach for the spread of cutaneous leishmaniasis disease in central Iran, Isfahan. Environ Modell Softw. 2016;82:330-46.

321. Randolph SE, Rogers DJ. Fragile transmission cycles of tick-borne encephalitis virus may be disrupted by predicted climate change. Proceedings Biological sciences. 2002;267:1741-4.

322. Ranjbar M, Shoghli A, Kolifarhood G, Tabatabaei SM, Amlashi M, Mohammadi M. Predicting factors for malaria re-introduction: an applied model in an elimination setting to prevent malaria outbreaks. Malaria Journal. 2016;15:12.

323. Rateb F, Merelo JJ, Arenas MG, Pavard B, Bellamine-BenSaoud N. The impact of education on healthcare: A malaria agent-based simulation. Modelling and Simulation 2003. 2003:378-82.

324. Reiner Jr RC, Stoddard ST, Scott TW. Socially structured human movement shapes dengue transmission despite the diffusive effect of mosquito dispersal. Epidemics. 2014;6:30-6.

325. Riedel N, Vounatsou P, Miller JM, Gosoniu L, Chizema-Kawesha E, Mukonka V, et al. Geographical patterns and predictors of malaria risk in Zambia: Bayesian geostatistical modelling of the 2006 Zambia national malaria indicator survey (ZMIS). 2010;9.

326. Riley S, Leung GM, Ho LM, Cowling BJ. Transmission of Japanese encephalitis virus in Hong Kong. Hong Kong Medical Journal. 2012;18:45-6.

327. Robert MA, Christofferson RC, Silva NJ, Vasquez C, Mores CN, Wearing HJ. Modeling mosquito-borne disease spread in U.S. urbanized areas: The case of dengue in Miami. PLoS One. 2016;11:e0161365.

328. Robinson S, Neitzel D, Moen R, Craft M, Hamilton K, Johnson L, et al. Disease risk in a dynamic environment: The spread of tick-borne pathogens in Minnesota, USA. EcoHealth. 2015;12:152-63.

329. Roche B, Léger L, L'Ambert G, Lacour G, Foussadier R, Besnard G, et al. The spread of *Aedes albopictus* in metropolitan France: Contribution of environmental drivers and human activities and predictions for a near future. PLoS ONE. 2015;10.

330. Rochlin I, Ninivaggi DV, Hutchinson ML, Farajollahi A. Climate change and range expansion of the Asian tiger mosquito (*Aedes albopictus*) in Northeastern USA: Implications for public health practitioners. Plos One. 2013;8:9.

331. Rocklöv J, Quam MB, Sudre B, German M, Kraemer MUG, Brady O, et al. Assessing seasonal risks for the introduction and mosquito-borne spread of Zika virus in Europe. EBioMedicine. 2016;9:250-6.

332. Roiz D, Neteler M, Castellani C, Arnoldi D, Rizzoli A. Climatic factors driving invasion of the tiger mosquito (*Aedes albopictus*) into new areas of Trentino, Northern Italy. Plos One. 2011;6:8.

333. Roy-Dufresne E, Logan T, Simon JA, Chmura GL, Millien V. Poleward expansion of the white-footed mouse (*Peromyscus leucopus*) under climate change: Implications for the spread of Lyme disease. Plos One. 2013;8:8.

334. Ruiz D, Poveda G, Vélez ID, Quiñones ML, Rúa GL, Velásquez LE, et al. Modelling entomological-climatic interactions of *Plasmodium falciparum* malaria transmission in two Colombian endemic-regions: Contributions to a National Malaria Early Warning System. Malaria Journal. 2006;5.

335. Ruiz MO, Chaves LF, Hamer GL, Sun T, Brown WM, Walker ED, et al. Local impact of temperature and precipitation on West Nile virus infection in *Culex* species mosquitoes in northeast Illinois, USA. Parasites & vectors. 2010;3:19.

336. Ruiz-Moreno D, Vargas IS, Olson KE, Harrington LC. Modeling dynamic introduction of chikungunya virus in the United States. Plos Neglected Tropical Diseases. 2012;6:8.

337. Ruktanonchai NW, DeLeenheer P, Tatem AJ, Alegana VA, Caughlin TT, Erbach-Schoenberg EZ, et al. Identifying malaria transmission foci for elimination using human mobility data. PLoS Comput Biol. 2016;12:19.

338. Ryden P, Sjostedt A, Johansson A. Effects of climate change on tularaemia disease activity in Sweden. Global health action. 2010;2.

339. Saad-Roy CM, van den Driessche P, Ma J. Estimation of Zika virus prevalence by appearance of microcephaly. 2016;16:754.

340. Sabatier P, Bicout DJ, Durand B, Dubois MA. Le recours a la modelisation en epidemiologie animale. Journee AEEMA: 19 Mai 2005. 2005:15-33.

341. Sabatier P, Dubois MA, Lancelot R, Hendrickx P, Lacaux JP. Space surveillance of epidemics: Case of the Rift Valley fever. Observing Our Environment for Space: New Solutions for a New Millennium. 2002:317-23.

342. Samat NA, Percy DF. Vector-borne infectious disease mapping with stochastic difference equations: An analysis of dengue disease in Malaysia. 2012;39:2029-46.

343. Samy AM, Elaagip AH, Kenawy MA, Ayres CF, Peterson AT, Soliman DE. Climate change influences on the global potential distribution of the mosquito *Culex quinquefasciatus*, vector of West Nile Virus and lymphatic filariasis. PLoS One. 2016;11:e0163863.

344. Samy AM, Thomas SM, Wahed AA, Cohoon KP, Peterson AT. Mapping the global geographic potential of Zika virus spread. Memorias do Instituto Oswaldo Cruz. 2016;111:559-60.

345. Sandoval-Ruiz CA, Zumaquero-Rios JL, Rojas-Soto OR. Predicting geographic and ecological distributions of triatomine species in the southern Mexican state of Puebla using ecological niche modeling. Journal of medical entomology. 2008;45:540-6.

346. Schäfer ML, Lundström JO. The present distribution and predicted geographic expansion of the floodwater mosquito *Aedes sticticus* in Sweden. Journal of Vector Ecology. 2009;34:141-7.

347. Schrader M, Hauffe T, Zhang Z, Davis GM, Jopp F, Remais JV, et al. Spatially explicit modeling of schistosomiasis risk in eastern China based on a synthesis of epidemiological, environmental and intermediate host genetic data. PLoS neglected tropical diseases. 2013;7:e2327.

348. Schröder W, Schmidt G. Spatial modelling of the potential temperature-dependent transmission of vector-associated diseases in the face of climate change: Main results and recommendations from a pilot study in Lower Saxony (Germany). Parasitology Research. 2008;103:S55-S63.

349. Schröder W, Schmidt G. Mapping the potential temperature-dependent tertian malaria transmission within the ecoregions of Lower Saxony (Germany). International Journal of Medical Microbiology. 2008;298:38-49.

350. Schur N, E H. Spatially explicit schistosoma infection risk in eastern Africa using Bayesian geostatistical modelling. Acta Trop. 2013.

351. Scoglio CM, Bosca C, Riad MH, Sahneh FD, Britch SC, Cohnstaedt LW, et al. Biologically informed individual-based network model for Rift Valley fever in the US and evaluation of mitigation strategies. Plos One. 2016;11:26.

352. Semenza JC, Tran A, Espinosa L, Sudre B, Domanovic D, Paz S. Climate change projections of West Nile virus infections in Europe: Implications for blood safety practices. Environmental Health: A Global Access Science Source. 2016;15:125-36.

353. Senay SD, Worner SP, Ikeda T. Novel three-step pseudo-absence selection technique for improved species distribution modelling. PLoS One. 2013;8:e71218.

354. Sewe MO, Ahlm C, Rocklov J. Remotely sensed environmental conditions and malaria mortality in three malaria Endemic Regions in Western Kenya. PLoS One. 2016;11:e0154204.

355. Shand L, Brown WM, Chaves LF, Goldberg TL, Hamer GL, Haramis L, et al. Predicting West Nile virus infection risk from the synergistic effects of rainfall and temperature. Journal of medical entomology. 2016;53:935-44.

356. Shearer FM, Huang Z, Weiss DJ, Wiebe A, Gibson HS, Battle KE, et al. Estimating geographical variation in the risk of zoonotic *Plasmodium knowlesi* infection in countries eliminating malaria. 2016;10:e0004915.

357. Signorini M, Cassini R, Drigo M, Frangipane di Regalbono A, Pietrobelli M, Montarsi F, et al. Ecological niche model of *Phlebotomus perniciosus*, the main vector of canine leishmaniasis in north-eastern Italy. Geospatial health. 2014;9:193-201.

358. Simon JA, Marrotte RR, Desrosiers N, Fiset J, Gaitan J, Gonzalez A, et al. Climate change and habitat fragmentation drive the occurrence of *Borrelia burgdorferi*, the agent of Lyme disease, at the northeastern limit of its distribution. Evol Appl. 2014;7:750-64.

359. Simpson JE, Hurtado PJ, Medlock J, Molaei G, Andreadis TG, Galvani AP, et al. Vector host-feeding preferences drive transmission of multi-host pathogens: West Nile virus as a model system. Proc R Soc B-Biol Sci. 2012;279:925-33.

360. Singh S, Shukla JB, Chandra P. Modelling and analysis of the spread of malaria: Environmental and ecological effects. Journal of Biological Systems. 2005;13:1-11.

361. Slater H, Michael E. Predicting the current and future potential distributions of lymphatic filariasis in Africa using maximum entropy ecological niche modelling. Plos One. 2012;7:14.

362. Slater H, Michael E. Mapping, Bayesian geostatistical analysis and spatial prediction of lymphatic filariasis prevalence in Africa. Plos One. 2013;8:14.

363. Snall T, Benestad RE, Stenseth NC. Expected future plague levels in a wildlife host under different scenarios of climate change. Global Change Biology. 2009;15:500-7.

364. Springer YP, Jarnevich CS, Barnett DT, Monaghan AJ, Eisen RJ. Modeling the present and future geographic distribution of the lone star tick, *Amblyomma americanum (Ixodida: Ixodidae*), in the continental United States. The American journal of tropical medicine and hygiene. 2015;93:875-90.

365. St John HK, Adams ML, Masuoka PM, Flyer-Adams JG, Jiang J, Rozmajzl PJ, et al. Prevalence, distribution, and development of an ecological niche model of *Dermacentor variabilis* ticks positive for *Rickettsia montanensis*. Vector-Borne and Zoonotic Diseases. 2016;16:253-63.

366. Stensgaard A, Jorgensen A, Kabatereine NB, Malone JB, Kristensen TK. Modeling the distribution of *Schistosoma mansoni* and host snails in Uganda using satellite sensor data and geographical information systems. Parassitologia. 2005;47:115-25.

367. Stensgaard AS, Booth M, Nikulin G, McCreesh N. Combining process-based and correlative models improves predictions of climate change effects on *Schistosoma mansoni* transmission in eastern Africa. Geospatial Health. 2016;11:94-101.

368. Stensgaard AS, Jørgensen A, Kabatereine NB, Rahbek C, Kristensen TK. Modeling freshwater snail habitat suitability and areas of potential snail-borne disease transmission in Uganda. Geospatial health. 2006;1:93-104.

369. Stensgaard AS, Utzinger J, Vounatsou P, Hurlimann E, Schur N, Saarnak CF, et al. Large-scale determinants of intestinal schistosomiasis and intermediate host snail distribution across Africa: Does climate matter? Acta Trop. 2011;128:378-90.

370. Subramanian A, Singh V, Sarkar RR. Understanding visceral leishmaniasis disease transmission and its control - A study based on mathematical modeling. Mathematics. 2015;3:913-44.

371. Sudheer C, Sohani SK, Kumar D, Malik A, Chahar BR, Nema AK, et al. A support vector machine-firefly algorithm based forecasting model to determine malaria transmission. Neurocomputing. 2014;129:279-88.

372. Supriatna AK, Anggriani N. The effect of recruitment rate and other demographic parameters on the transmission of dengue disease. Symposium on Biomathematics. 2015;1651:128-37.

373. Sweeney AW, Beebe NW, Cooper RD, Bauer JT, Peterson AT. Environmental factors associated with distribution and range limits of malaria vector *Anopheles farauti* in Australia. Journal of medical entomology. 2006;43:1068-75.

374. Tachiiri K, Klinkenberg B, Mak S, Kazmi J. Predicting outbreaks: A spatial risk assessment of West Nile virus in British Columbia. International journal of health geographics. 2006;5:21.

375. Talla C, Diallo D, Dia I, Ba Y, Ndione JA, Sall AA, et al. Statistical modeling of the abundance of vectors of West African Rift Valley fever in Barkedji, Senegal. Plos One. 2014;9:23.

376. Tanser FC, Sharp B, Sueur Dl. Potential effect of climate change on malaria transmission in Africa. 2003;362:1792-8.

377. Tatem AJ, Hay SI, Rogers DJ. Global traffic and disease vector dispersal. Proceedings of the National Academy of Sciences. 2006.

378. Taylor D, Hagenlocher M, Jones AE, Kienberger S, Leedale J, Morse AP. Environmental change and Rift Valley fever in eastern Africa: Projecting beyond Healthy Futures. Geospatial Health. 2016;11:115-28.

379. Teklehaimanot HD, Lipsitch M, Teklehaimanot A, Schwartz J. Weather-based prediction of *Plasmodium falciparum* malaria in epidemic-prone regions of Ethiopia I. Patterns of lagged weather effects reflect biological mechanisms. Malaria journal. 2004;3:41.

380. Thomas SM, Tjaden NB, van den Bos S, Beierkuhnlein C. Implementing cargo movement into climate based risk assessment of vector-borne diseases. International Journal of Environmental Research and Public Health. 2014;11:3360-74.

381. Thomson MC, Elnaiem DA, Ashford RW, Connor SJ. Towards a kala azar risk map for Sudan: Mapping the potential distribution of *Phlebotomus orientalis* using digital data of environmental variables. Tropical medicine & international health : TM & IH. 1999;4:105-13.

382. Tompkins DM, Slaney D. Exploring the potential for Ross River virus emergence in New Zealand. Vector borne and zoonotic diseases (Larchmont, NY). 2014;14:141-8.

383. Tonnang HE, Kangalawe RY, Yanda PZ. Predicting and mapping malaria under climate change scenarios: The potential redistribution of malaria vectors in Africa. Malaria journal. 2010;9:111.

384. Trájer A, Bede-Fazekas Á, Bobvos J, Páldy A. Seasonality and geographical occurrence of West Nile fever and distribution of Asian tiger mosquito. Idojaras. 2014;118:19-40.

385. Trajer AJ, Bede-Fazekas A, Hufnagel L, Horvath L, Bobvos J, Paldy A. The effect of climate change on the potential distribution of the European *Phlebotomus* species. 2013;11:189-208.

386. Tran A, Ippoliti C, Balenghien T, Conte A, Gely M, Calistri P, et al. A geographical information system-based multicriteria evaluation to map areas at risk for Rift Valley fever vector-borne transmission in Italy. Transboundary and Emerging Diseases. 2013;60:14-23.

387. Tran A, L'Ambert G, Lacour G, Benoît R, Demarchi M, Cros M, et al. A rainfall- and temperature-driven abundance model for *Aedes albopictus* populations. International Journal of Environmental Research and Public Health. 2013;10:1698-719.

388. Tran A, Raffy M. On the dynamics of dengue epidemics from large-scale information. Theoretical population biology. 2005;69:3-12.

389. Tran A, Sudre B, Paz S, Rossi M, Desbrosse A, Chevalier V, et al. Environmental predictors of West Nile fever risk in Europe. International journal of health geographics. 2014;13:26.

390. Tran A, Trevennec C, Lutwama J, Sserugga J, Gély M, Pittiglio C, et al. Development and assessment of a geographic knowledge-based model for mapping suitable areas for Rift Valley fever transmission in Eastern Africa. PLoS Neglected Tropical Diseases. 2016;10.

391. Vaidya A, Bravo-Salgado AD, Mikler AR. Modeling climate-dependent population dynamics of mosquitoes to guide public health policies. ACM BCB 2014 - 5th ACM Conference on Bioinformatics, Computational Biology, and Health Informatics. 2014:380-9.

392. Valiakos G, Papaspyropoulos K, Giannakopoulos A, Birtsas P, Tsiodras S, Hutchings MR, et al. Use of wild bird surveillance, human case data and GIS spatial analysis for predicting spatial distributions of West Nile virus in Greece. Plos One. 2014;9:8.

393. van Lieshout M, Kovats RS, Livermore MTJ, Martens P. Climate change and malaria: Analysis of the SRES climate and socio-economic scenarios. Global Environmental Change Part A: Human & Policy Dimensions. 2004;14:87.

394. Vanwambeke S, Lambin E, Eichhorn M, Flasse S, Harbach R, Oskam L, et al. Impact of land-use change on dengue and malaria in Northern Thailand. EcoHealth. 2007;4:37-51.

395. Vega GJdl, Medone P, Ceccarelli S, Rabinovich J, Schilman PE. Geographical distribution, climatic variability and thermo-tolerance of Chagas disease vectors. 2015;38:851-60.

396. Villela DAM, Codeço CT, Figueiredo F, Garcia GA, Maciel-de-Freitas R, Struchiner CJ. A Bayesian hierarchical model for estimation of abundance and spatial density of *Aedes aegypti*. PLoS ONE. 2015;10.

397. Waite JL, Lynch PA, Thomas MB. Eave tubes for malaria control in Africa: A modelling assessment of potential impact on transmission. Malaria Journal. 2016;15:10.

398. Walsh M, Haseeb MA. Modeling the ecologic niche of plague in sylvan and domestic animal hosts to delineate sources of human exposure in the western United States. PeerJ. 2015;3:e1493.

399. Walter KS, Pepin KM, Webb CT, Gaff HD, Krause PJ, Pitzer VE, et al. Invasion of two tick-borne diseases across New England: Harnessing human surveillance data to capture underlying ecological invasion processes. Proc R Soc B-Biol Sci. 2016;283:10.

400. Wang HH, Grant WE, Teel PD, Hamer SA. Tick-borne infectious agents in nature: Simulated effects of changes in host density on spatial-temporal prevalence of infected ticks. Ecological Modelling. 2016;323:77-86.

401. Wangdi K, Singhasivanon P, Silawan T, Lawpoolsri S, White NJ, Kaewkungwal J. Development of temporal modelling for forecasting and prediction of malaria infections using time-series and ARIMAX analyses: A case study in endemic districts of Bhutan. Malaria journal. 2010;9:251.

402. Wardrop NA, Atkinson PM, Gething PW, Fevre EM, Picozzi K, Kakembo ASL, et al. Bayesian geostatistical analysis and prediction of Rhodesian human African trypanosomiasis. Plos Neglected Tropical Diseases. 2010;4:10.

403. Wesolowski A, Eagle N, Tatem AJ, Smith DL, Noor AM, Snow RW, et al. Quantifying the impact of human mobility on malaria. Science. 2012.

404. Wesolowski A, Qureshi T, Boni MF, Sundsøy PR, Johansson MA, Rasheed SB, et al. Impact of human mobility on the emergence of dengue epidemics in Pakistan. Proceedings of the National Academy of Sciences of the United States of America. 2015;112:11887-92.

405. White MT, Shirreff G, Karl S, Ghani AC, Mueller I. Variation in relapse frequency and the transmission potential of *Plasmodium vivax* malaria. Proc R Soc B-Biol Sci. 2016;283:9.

406. Williams CR, Gill BS, Mincham G, Mohd Zaki AH, Abdullah N, Mahiyuddin WR, et al. Testing the impact of virus importation rates and future climate change on dengue activity in Malaysia using a mechanistic entomology and disease model. Epidemiology and infection. 2015;143:2856-64.

407. Williams CR, Mincham G, Faddy H, Viennet E, Ritchie SA, Harley D. Projections of increased and decreased dengue incidence under climate change. Epidemiology and infection. 2016;144:3091-100.

408. Williams CR, Mincham G, Ritchie SA, Viennet E, Harley D. Bionomic response of *Aedes aegypti* to two future climate change scenarios in far north Queensland, Australia: implications for dengue outbreaks. Parasites & vectors. 2014;7:447.

409. Williams CR, Ritchie SA, Whelan PI. Potential distribution of the Asian disease vector *Culex gelidus Theobald (Diptera: Culicidae*) in Australia and New Zealand: A prediction based on climate suitability. 2005;44:425-30.

410. Williams HW, Cross DE, Crump HL, Drost CJ, Thomas CJ. Climate suitability for European ticks: Assessing species distribution models against null models and projection under AR5 climate. Parasites & vectors. 2015;8:440.

411. Wimberly MC, Baer AD, Yabsley MJ. Enhanced spatial models for predicting the geographic distributions of tick-borne pathogens. International journal of health geographics. 2008;7:15.

412. Winters AM, Staples JE, Ogen-Odoi A, Mead PS, Griffith K, Owor N, et al. Spatial risk models for human plague in the West Nile region of Uganda. The American journal of tropical medicine and hygiene. 2009;80:1014-22.

413. Wu X, Röst G, Zou X. Impact of spring bird migration on the range expansion of *Ixodes scapularis* tick population. Bulletin of Mathematical Biology. 2016;78:138-68.

414. Xue L, Cohnstaedt LW, Scott HM, Scoglio C. A Hierarchical network approach for modeling Rift Valley fever epidemics with applications in North America. Plos One. 2013;8:12.

415. Yaacob Y, Yeak SH, Lim RS, Soewono E. A delay differential equation model for dengue transmission with regular visits to a mosquito breeding Site. Symposium on Biomathematics. 2015;1651:153-60.

416. Yamana TK, Eltahir EA. Early warnings of the potential for malaria transmission in rural Africa using the hydrology, entomology and malaria transmission simulator (HYDREMATS). Malaria journal. 2010;9:323.

417. Yamana TK, Eltahir EA. Projected impacts of climate change on environmental suitability for malaria transmission in West Africa. Environmental health perspectives. 2013;121:1179-86.

418. Yang GJ, Tanner M, Utzinger J, Malone JB, Bergquist R, Yy Chan E, et al. Malaria surveillance-response strategies in different transmission zones of the People's Republic of China: Preparing for climate change. Malaria Journal. 2012;11.

419. Yang HM, Ferreira MU. Assessing the effects of global warming and local social and economic conditions on the malaria transmission. Revista de saude publica. 2000;34:214-22.

420. Yang K, Wang X, Yang G, Wu X, Qi Y, Li H, et al. An integrated approach to identify distribution of *Oncomelania hupensis*, the intermediate host of *Schistosoma japonicum*, in a mountainous region in China. 2008;38:1007-16.

421. Yé Y, Hoshen M, Kyobutungi C, Louis VR, Sauerborn R. Local scale prediction of *Plasmodium falciparum* malaria transmission in an endemic region using temperature and rainfall. Global Health Action. 2009;2.

422. Yiannakoulias NW, Svenson LW. West Nile virus: Strategies for predicting municipal-level infection. Annals of the New York Academy of Sciences. 2007;1102:135-48.

423. Yoo EH. Site-specific prediction of West Nile virus mosquito abundance in Greater Toronto Area using generalized linear mixed models. 2014;28:296-313.

424. Yoo EH, Trawinski PR. Joint space-time modeling of West Nile virus vector mosquito abundance. Accuracy 2010 - Proceedings of the 9th International Symposium on Spatial Accuracy Assessment in Natural Resources and Environmental Sciences. 2010:193-5.

425. York EM, Butler CJ, Lord WD. Global decline in suitable habitat for *Angiostrongylus ( = Parastrongylus) cantonensis*: The role of climate change. PLoS One. 2014;9:e103831.

426. Yu HL, Yang SJ, Yen HJ, Christakos G. A spatio-temporal climate-based model of early dengue fever warning in southern Taiwan. Stochastic Environmental Research and Risk Assessment. 2011;25:485-94.

427. Zhang Z, Ong S, Peng W, Zhou Y, Zhuang J, Zhao G, et al. A model for the prediction of *Oncomelania hupensis* in the lake and marshland regions, China. Parasitology international. 2008;57:121-31.

428. Zhou X, Yang G, Yang K, Wang X, Hong Q, Sun L, et al. Potential impact of climate change on schistosomiasis transmission in China. 2008;78:188-94.
